# *H2r*: Identification of evolutionary important residues by means of an entropy based analysis of multiple sequence alignments

**DOI:** 10.1186/1471-2105-9-151

**Published:** 2008-03-18

**Authors:** Rainer Merkl, Matthias Zwick

**Affiliations:** 1 Institut für Biophysik und Physikalische Biochemie, Universität Regensburg, D-93040 Regensburg, Germany; 2 Biozentrum, Universität Basel, CH-4056 Basel, Switzerland

## Abstract

**Background:**

A multiple sequence alignment (MSA) generated for a protein can be used to characterise residues by means of a statistical analysis of single columns. In addition to the examination of individual positions, the investigation of co-variation of amino acid frequencies offers insights into function and evolution of the protein and residues.

**Results:**

We introduce *conn(k)*, a novel parameter for the characterisation of individual residues. For each residue *k*, *conn(k) *is the number of most extreme signals of co-evolution. These signals were deduced from a normalised mutual information (*MI*) value *U*(*k*, *l*) computed for all pairs of residues *k*, *l*. We demonstrate that *conn(k) *is a more robust indicator than an individual *MI*-value for the prediction of residues most plausibly important for the evolution of a protein. This proposition was inferred by means of statistical methods. It was further confirmed by the analysis of several proteins. A server, which computes *conn(k)*-values is available at .

**Conclusion:**

The algorithms *H2r*, which analyses MSAs and computes *conn(k)*-values, characterises a specific class of residues. In contrast to strictly conserved ones, these residues possess some flexibility in the composition of side chains. However, their allocation is sensibly balanced with several other positions, as indicated by *conn(k)*.

## Background

Without any doubt, a multiple sequence alignment (MSA) offers a wealth of information about the related protein. Most easily, conserved residues can be identified, which indicate positions crucial for function or structure. Therefore, MSAs are frequently the basis for the prediction of important residues; see *e.g. *[1,2]. For quantification of residue conservation several scores have been introduced; for a review see [3]. Even more effort needs the recognition of those residues, which are not strictly conserved, but depend on the composition of their neighbourhood. In the simplest case, two contacting residues may show a strictly coupled occurrence of amino acids. In addition to direct contacts, several, less obvious reasons like the concerted instrumentation of active sites or the signalling of allostery may be responsible for dependencies in amino acid frequencies. In summary, the co-evolution of residues is expected to induce patterns detectable by correlation analysis. Knowing these correlations does help to characterise more residues, may facilitate the understanding of protein function, and implies additional constraints to be considered in protein design and mutagenesis experiments. The importance of these signals and their consequences have long been realised [4] and quite different approaches have been introduced to identify correlated pairs. Neher has adapted a method developed to analyse noisy patch clamp signals [5]. Göbel *et al. *have determined a correlation coefficient [6]. The mutual information content of residue pairs [7,8] or chi-squared statistical methods have been exploited [9]. A similar approach has been used to map allosteric communication in GroEL [10]. In order to enhance the quality of the prediction, additional parameters like alignment stability have been utilised [11].

The above methods rely on the computation of a global co-variation statistic for the identification of correlated residues. In contrast to these concepts, methods based on the idea of "perturbations" have been introduced recently [12,13]. An *in silico *perturbation is a constraint that limits the occurrence of amino acids at a certain position. Each choice selects a specific subset of MSA sequences and may cause variations in the column-specific occurrence of amino acids. Analysing these patterns, Ranganathan and co-workers have proposed the existence of energetically coupled residues [14]. A similar algorithm has been applied to identify networks that regulate allostery [15]. In combination with molecular dynamics, perturbation analysis has been used to predict residues essential for catalysis [16].

The enormous increase of sequence information resulting from genome sequencing projects has broadened the data basis for coupling analysis. Therefore, methods can be used that examine a large number of parameters. Even more, the existence of a high quality MSA is crucial for the analysis of correlated mutations. The sequence space of a protein has to be sampled correctly; otherwise, the quality of the predictions will deteriorate. If similar sequences originating from closely related species majorise an MSA, signals caused by a shared evolution of the proteins may be stronger than correlation patterns. Such bias will influence any calculation. However, methods based on the analysis of perturbations may be susceptible to less accented distortions. In this case, smaller sets of sequences determine predictions and may constitute signals interpreted as perturbations. If these subalignments are dominated by closely related sequences, the predictions may be wrong.

This is why we prefer algorithms exploiting exhaustively the information deposited in each column of an MSA. In the following, we report *H2r*, a novel algorithm of that kind. *H2r *combines classical and well-proven concepts of computer science. It was our aim to focus on reliability even at the expense of sensitivity. We will confirm *H2r*'s robustness and show that coupled residue pairs identified by *H2r *constitute tightly interconnected networks. Parameters will be introduced that allow the characterisation of these networks and individual residues. It will be demonstrated that the mode of generating MSAs does not markedly influence *H2r*'s results. We study predictions in protein 3D-space and discuss possible reasons for the evolution of correlation patterns.

## Results

### The measure *U*(*k*, *l*) exploits all the association preserved in columns of MSAs

A large number of algorithms, utilising quite different principles have been introduced to identify correlated mutations. The co-variance algorithm proposed in [12] uses the concept of perturbations for the identification of coupled residues. In order to identify perturbations, all those positions *k *have to be found, where an amino acid $a_{i}^{k}$ = *X *occurs with a certain minimal frequency *f*_min_($a_{i}^{k}$). For those sequences possessing *X *at position *k*, the conditional frequencies $f(a_{j}^{l}|a_{i}^{k}=X)$ at all other positions *l *have to be determined and compared *e.g. *to mean frequencies $f(a_{j}^{l})$. However, this approach does not fully exploit the information given by the MSA as only one set of conditional probabilities is analysed for each column. If (say) one amino acid *X *is represented in 40% of the sequences at position *k*, 60% of the information embedded in columns *k *and *l *is left without interpretation. This is also true for the algorithm introduced in [13], which uses likelihood values. To overcome this drawback we propose *U*(*k*, *l*), which originates from Shannon's information theory [17] and is closely related to an approach that has been introduced recently [8]. Formally, Shannon's concepts are similar to Boltzmann's statistical mechanics. However, these ideas are solely based on probabilities and do not need an interpretation as energy terms.

A parameter frequently used for quantifying the composition of an individual column *k *is its entropy *H*(*k*); see [18] and references therein:

(1) $$H(k)=-\sum_{i=1}^{20}f(a_{i}^{k})\ln\;f(a_{i}^{k})$$

Here, *f*($a_{i}^{k}$) is the frequency of amino acid *a*_*i *_at position *k*. Please note that we use frequencies instead of probabilities. We consider the MSA as a representative sample allowing the estimation of all the parameters we need.

The entropy *H*(*k*, *l*) of two variables (columns) *k *and *l *is

(2) $$H(k,l)=-\sum_{i,j}f(a_{i}^{k},a_{j}^{l})\ln\;f(a_{i}^{k},a_{j}^{l})$$

Using formulas (1) and (2), the mutual information *MI*(*k*, *l*) = *H*(*k*) + *H*(*l*) - *H*(*k*, *l*) can be computed. *MI*-values have been the basis for several analyses. However, it has been shown that raw *MI*-values are a poor indicator for the prediction of co-evolution [8]. More reliable are normalised *MI*-values. For synthetic MSAs, the ratios *MI*(*k*, *l*)/*H*(*k*, *l*) or *MI*(*k*, *l*)/(*H*(*k*) + *H*(*l*)) have performed best [8]. In the following, we use the parameter *U*(*k*, *l*), which is a measure for the dependency of *k *on *l *and *vice versa*:

(3) $$U(k,l)=2\frac{H(k)+H(l)-H(k,l)}{H(k)+H(l)}$$

It follows that 0 ≤ *U*(*k*, *l*) ≤ 1.0: If columns *k *and *l *are completely independent, then *H*(*k*, *l*) = *H*(*k*) + *H*(*l*) and *U*(*k*, *l*) vanishes. If the two columns are completely dependent, then *H*(*k*) = *H*(*l*) = *H*(*k*, *l*) and *U*(*k*, *l*) equals 1.0. For the analysis of correlated mutations in MSAs, high values of *U*(*k*, *l*) indicate a strict pair-wise co-occurrence of amino acids in columns *k *and *l*. In more detail, formula (3) has been discussed in [19], which comprises an implementation, too. *H*(*k*, *l*) can directly be deduced from frequencies $f(a_{i}^{k},a_{j}^{l})$, which have to be determined for all *i *= 1..20, all *j *= 1..20 amino acids, and all combinations of positions *k *and *l*. This implies that the MSA has to be large enough to allow a reliable estimation of these frequencies. For a similar approach, a lower limit of approximately 125 sequences has been determined [8]. For synthetic MSAs, we have shown that *U*(*k*, *l*)-values range as expected; see Additional File 1.

### *conn(k)*, a novel parameter for the characterisation of individual residues

To begin with, the outcome of a mutational analysis identifies coupled residue pairs *k*, *l*. Additionally, these values allow the assessment of individual positions *k*. For the ATP synthase *ε *subunit of *Escherichia coli*, it has been made plausible that residues with highest Z-scores deduced from normalised *MI*-values are more likely to change the activity than those with low values [20]. However, an individual score may be misleading. The risk of misclassification increases for weaker signals, *i.e. *for lower *U*(*k*, *l*)-values. Merely by chance and due to random fluctuations, residue pairs might be assigned a relatively large value resulting in a strikingly high Z-score.

**Figure 1:**
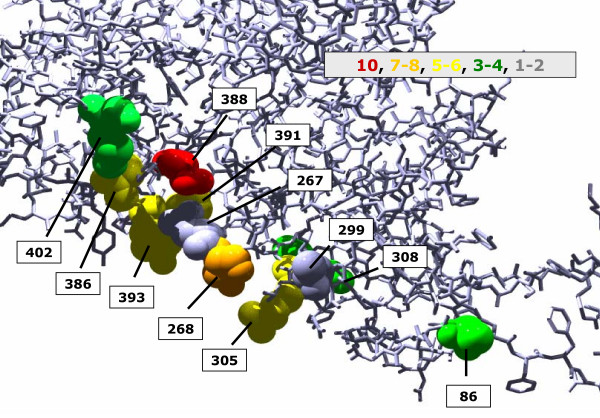
**Highest scoring residues *k *of **1QGN** interlinked with position 388**. All residues *k*, which are an element of the 10 highest scoring pairs (388, *k*), were plotted in space filling mode and labelled. The colour code indicates the magnitude of their *conn(k)*-values.

For any noisy signal, the quality of a prediction can be enhanced by sampling, *i.e. *by adding up several analyses. We applied this principle for the identification of conspicuous positions. In agreement with previous findings [20], high scoring residue pairs identified by *H2r *form tightly connected networks; see Additional File 1. Therefore, we utilised concepts of network analysis for the assessment of residues. A commonly used parameter that allows a characterisation of individual nodes within a network is their connectivity; see [21] and references therein. Here, we define the connectivity *conn(k) *as the number of high-scoring pairs a residue *k *is an element of. Connectivity values differ significantly: For the MSA associated with the PFAM [22] entry PF01053, *conn*(388) was 10 and *conn*(111) was 1. In order to illustrate how networks of interconnected residues are located in 3D-space, Figure 1 indicates those residues contributing to the *conn*(388)-value. The MSA of PF01053 (Cys_Met_Meta) and the related protein structure (pdb-code 1QGN) have already been a test bed for *in silico *analysis [13]. A further example, which supports the *conn(k)*-approach is the SH3 domain: For a chi-squared approach it has been shown that 5 residues participate in 53 of 92 significant co-variations [9].

In order to assess the parameter *conn(k) *in detail, we examined the outcome of *H2r *on several datasets. The first two experiments were carried out to estimate the probability of *conn(k)*-values for MSAs bearing no coupling signals of real proteins.

For parameter optimisation (see Additional File 1), we compiled a set containing 20 PFAM entries, which we named *H2r_train*. We used *H2r *to determine the occurrence and frequency of *conn(k)*-values for these MSAs. However, for the following assessment we had randomly assigned *U*(*k*, *l*)-values in the range of 0.0 to 1.0 to all pairs *k*, *l*. For this test, 500 individual experiments (one MSA each) were analysed. A second test was based on PF01053 that was introduced above. Here, we did 1000 independent experiments by assigning *U*(*k*, *l*)-values randomly and analysing the distribution of *conn(k)*-values. Results of both experiments are summarised in Table 1. Altogether, the experiments indicate that *conn(k)*-values ≥ 4 are highly unlikely to occur merely by chance. The frequency for *conn(k) *= 4 is < 2.5·10^-3^, a connectivity *conn(k) *> 6 was not observed in any of these experiments. Please note that only the 75 largest *U*(*k*, *l*)-values were analysed for each MSA. This has to be considered when interpreting the above frequencies.

**Table 1:** Frequency of *conn(k)*-values resulting from randomly assigned *U*(*k*, *l*)-values

	***conn(k)***					
	
	1	2	3	4	5	6
***H2r_train *(500 samples)**	0.82	0.16	0.02	2.4·10^-3^	2.6·10^-4^	3.2·10^-5^
**PF01053 (1000 samples)**	0.90	0.10	4.2·10^-3^	1.3·10^-4^	-	-

The datasets *H2r_train*, consisting of 20 randomly selected PFAM entries (see Additional file 1, Table S2) and PF01053 were analysed as explained. However, *U*(*k*, *l*)-values were assigned randomly. For *H2r_train*, 500 MSAs were analysed; for PF01053, 1000 experiments were done. For all experiments, the number of residues possessing the respective *conn(k)*-values was determined. The table lists frequency values deduced from the analysis of 75 *HSRPs *per sample.

**Figure 2:**
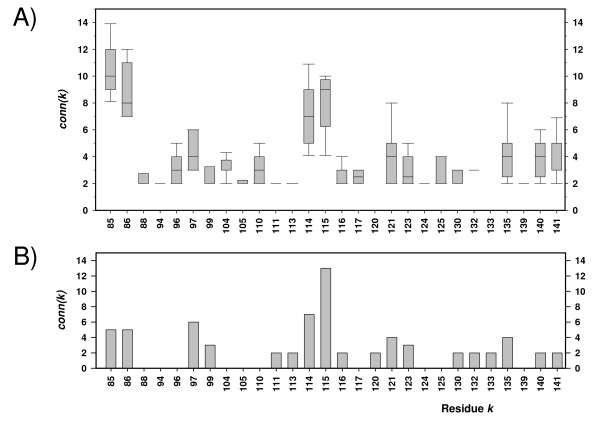
***Conn(k)*-values for PF00018**. PF00018 comprises 3506 sequences, which are arranged in a single MSA. 20 subsets were generated by selecting randomly 210 sequences in each case. Boxplots resulting from *conn(k)*-values ≥ 2 are summarised in Panel A. Panel B shows the predictions with *conn(k) *≥ 2 for the dataset *SH3_filt*, which contains 471 sequences and was created by filtering PF00018 with default filter values.

As a further test for the robustness of our approach, we analysed PF00018. This dataset subsumes 3506 sequences of SH3 domains. The domain consists of approximately 60 residues occurring in a large number of eukaryotic proteins involved in signal transduction. The 3D-structure of the related Fyn domain (a Src family tyrosine kinase; pdb-code 1SHF) has been determined [23]. Co-variation analysis has been utilised to predict tertiary contacts and to design compensating hydrophobic core substitutions [9]. Applying our default filter criteria (see Additional File 1) resulted in the dataset *SH3_filt*, which consisted of 471 sequences. Its *U*(*k*, *l*)-values were relatively low, the largest one, *U(85,114) *was 0.28. This observation indicates that these correlations are much weaker than those observed in Cys_Met_Meta, which possesses a maximal *U*(*k*, *l*)-value of 0.72. As PF00018 contains 3506 sequences, we used a bootstrapping approach to repeat the experiment several times and to analyse the results statistically. We generated 20 datasets by randomly selecting 210 sequences in each case. The results are summarised in Figure 2. Residues 85, 86, 97, 114, and 115 possess the five highest mean connectivity values; see Panel A. This finding corresponded to the *SH3_filt *results (compare Panel B) and proposed to accept *conn(k)*-values ≥ 5. Furthermore, this cut-off was supported by the following correspondences of function and *conn(k)*-values.

According to [23], the following structural and functional features of the SH3 domain are relevant to interpret the above results: A patch of aromatic residues is flanked by two loops: the n-Src loop (Ser115, Glu116) and the RT-Src loop (Arg96, Thr97). The ligand binding properties of the aromatic surface could be modulated by the residues of the RT-Src and the n-Src loop. Residues 85 and 86 are presumably important for linking the domain to the rest of the protein. Please note the correspondence of function and high connectivity values for 4 out of 5 predictions generated by *H2r*.

However, the extreme variation of *conn(k)*-values occurring for individual datasets of the bootstrapping approach made clear that the filtering of the input sequences is a critical step. A random selection of 210 sequences resulted *e.g. *for residue 135 in *conn(k)*-values ranging from 2 to 8.

The maximal *U*(*k*, *l*)-value determined for an MSA indicates the strength of the coupling signal. Our analysis of synthetic MSAs allows a rough estimation of the values; see Additional File 1. For Cys_Met_Meta, this maximum was 0.72, which is quite high. Above, we made plausible that a *conn(k)*-value ≥ 4 can be considered reliable in this case. For PF00018, the maximal *U*(*k*, *l*)-value was 0.28. For this dataset, biochemical findings allowed us to explain the role of residues with *conn(k)*-values ≥ 5. As a (conservative) rule of thumb, we propose a cut-off of 0.5: If the maximal *U*(*k*, *l*)-value is ≥ 0.5, *H2r *lists residues with *conn(k)*-values ≥ 4 otherwise those ≥ 5.

The above random sampling of sequences without any filtering induced a large variation of *conn(k)*-values. In order to characterise the outcome of *H2r *for a sampling on filtered data, we created datasets by randomly choosing 75% or 60% of the remaining sequences. Resulting *conn(k)*-values showed that the composition of these MSAs did not markedly affect *H2r*'s results (see Additional File 1). After applying our filtering procedure, the set of residues with highest *conn(k)*-values remained stable.

### *conn(k) *is more robust to random noise than individual *MI*-values

The ATP synthase *ε *subunit of *E. coli *has been extensively mutated and the effects of mutations have been characterised and compiled (see [20] and references therein). For an *in silico *analysis, a specific MSA has been generated and normalised *MI*-values have been used to compute Z-scores [20]. For each residue, the largest Z-score has been determined and compared to the above list of mutational effects. The authors have concluded that positions with high maximum Z-scores are more likely to change the activity of the protein upon mutagenesis than positions with a low score. We analysed the corresponding PF02823 and projected the results onto the structure deposited in pdb-file 1AQT. In addition, we computed a normalised *MI*-value as has been used previously [20] and determined the ranks of corresponding values. Detailed results are compiled in Additional file 1, Table S9. For this dataset, the ranks of maximal *U*(*k*, *l*)-values and normalised *MI*-values were identical for all high scoring residues. This indicates that both parameters allow equally well to quantify the coupling of residues. The maximal *U*(*k*, *l*)-value was 0.37 in this case. Therefore, *H2r *considered *conn(k)*-values ≥ 5 as reliable. This was true for positions 12, 65, 72, and 81. For positions 65 and 81 their susceptibility to mutational effects is known, none has been reported for the remaining two positions. If the four largest *U*(*k*, *l*)-values were used for predicting conspicuous residues *k*, a comparison of this approach and the *conn(k) *method gave the following result: In both cases, 2 positions known to be susceptible to mutational effects were predicted correctly. It is unknown, how mutations affect the other two residues. Thus, if one utilises the concordance with known mutational effects as an indicator for prediction quality, both approaches have a similar performance.

However, if the coupling signals were less pronounced the number of unclear predictions increased drastically for the maximum score approach. In Table 2, we have compared *conn(k) *and the maximal *U*(*k*, *l*)-value deduced from PF00018 representing the SH3 domain. The largest *U*(*k*, *l*)-value is 0.28. For none of the extra residues possessing maximum *U*(*k*, *l*)-values ranked 2 or below 3, a clear function has been reported in [23]. In summary, these findings indicate that *conn(k)*-values are more robust and more reliable than individual *U*(*k*, *l*)-scores.

**Table 2:** Analysis of SH3 domain

***#***	***Conn(k)***	***Max U(k, l)***
115	13 (1)	0.25 (3)
114	7 (2)	0.28 (1)
97	6 (3)	0.25 (3)
85	5 (4)	0.28 (1)
86	5 (4)	0.23 (10)
100		0.26 (2)
99		0.26 (2)
113		0.25 (4)
121		0.25 (4)
103		0.24 (5)
89		0.24 (5)
130		0.24 (7)
141		0.23 (10)

The MSA constituting PF00018 was analysed by using *H2r*. Residue numbers resulting from a projection of the MSA onto pdb structure 1SHF are listed in the first column. The second column lists *conn(k)*-values, the third one the maximal *U*(*k*, *l*)-values. The rank of each prediction is given in brackets.

Interestingly, residues 72 and 73 of the ATP synthase *ε *subunit had in both of the above experiments high *MI*-values; however no high Z-score has been reported [20]. This difference is most probably due to variations in the composition of the underlying MSAs.

### *conn(k)*-values characterise a so far unidentified group of residues

In order to compare the output of *H2r *to a perturbation based method, we analysed the MSA of globin sequences, as compiled in [14]. For this dataset, 32 residues have been predicted to constitute a network of allosteric communication. Applying our standard procedure, the largest *U*(*k*, *l*)-value was 0.72. 9 residues gained a *conn(k)*-value ≥ 4. These were – projected onto 2DN1 – residues **97**, 40, **57**, **93**, 131, 37, 85, 2, 39. Only 3 of these predictions (printed in bold) were in agreement with previous findings as reported in [14].

For the following comparisons, we used the above introduced proteins represented by a PFAM entry and a related protein structure: Cys_Met_Meta (PF01053, 1QGN) [13], SH3 domain (PF00018, 1SHF) [9], and ATP synthase *ε *subunit (PF02823, 1AQT) [20]. We selected these proteins, as all three have been studied extensively by means of *in silico *methods. Please note that we did not compile specific MSAs but used the precompiled full PFAM alignments for the following tests.

The server based on [10,24] did not find correlated mutations for PF00018, PF01053, and PF02823. P2PConPred is a server for the prediction of residue-residue contacts and residue correlations [25], which exploits pair-to-pair amino acid substitution matrices deduced from high quality alignments. For each of the above datasets, we selected a full PFAM alignment, used default parameters, and projected the results onto the above structures offered by the server. None of the predictions was a *HSRP *(data not shown). CorrMut is a server identifying correlations in the evolution of amino acid sequences [26]. After selecting a pdb-file as input, it returns a list of correlated residue pairs. The analysis of the above structures contained no *HSRP *in all three cases (data not shown).

Based on a chi-squared statistical method, the 25 top co-varying SH3 residues have been computed and ranked [9]. Our results did not coincide with these findings; the following ranks (given in brackets) have been assigned to the residues predicted by *H2r*: 85 (15), 86 (-), 97 (-), 114 (22), 115 (-). Thus, *H2r *did not confirm any of the 14 top ranking predictions. Interestingly, our implementation of a Göbel like algorithm [6] assigned highest connectivity values to residues 91 (6), 92 (-), 93 (-), 98 (-), and 100 (11). Again, the ranks that are given in brackets demonstrate that these results do not coincide with the findings of the chi-squared test. In summary, these findings made clear that *MI*-based methods like *H2r *and the above algorithms differ quite significantly in their predictions. This statement is further supported by an analysis of a larger dataset reported in the Additional File 1.

### Predicting co-evolving residues for enzymes of tryptophan synthesis

**Figure 3:**
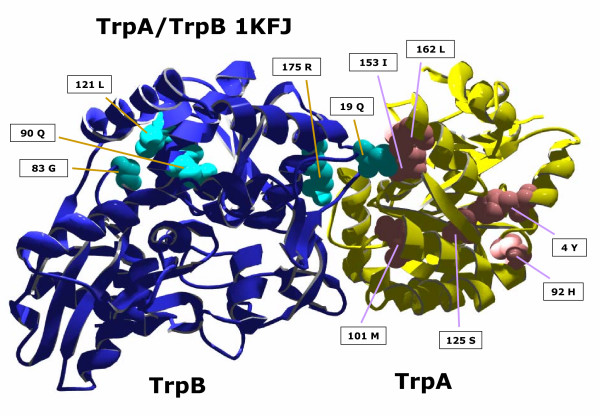
**Residues of the TrpA/TrpB complex possessing highest *conn(k)*-values**. For TrpA and TrpB (pdb-code 1KFJ), residues with *conn(k)*-values ≥ 4 were plotted in space filling mode and labelled. For TrpA *conn*(162) = 8 and for TrpB *conn*(90) = 8 were the highest values.

As illustrative examples, we analysed three enzymes of the tryptophan synthesis pathway. TrpA and TrpB constitute the *αββα *tryptophan synthase complex, which catalyses the final reaction from indole-3-glycerole phosphate + L-serine to L-tryptophan + H_2_O. The *α *subunit (TrpA) cleaves indoleglycerol-3-phosphate to glyceraldehyde-3-phosphate and indole. The latter is transported through a hydrophobic tunnel to the associated *β *subunit (TrpB), where it is condensed with L-serine to yield L-tryptophan. A sophisticated mechanism of allostery links the *α *and *β *monomers of the synthase[27]. Both proteins share a common evolution [28]. In Figure 3, the predictions of *H2r *for TrpA and TrpB are plotted as projected onto pdb-entry 1KFJ [29]. For TrpA, 6 conspicuous residues have been identified. Residue 162, which possesses the largest *conn(k)*-value, is an element of the TrpA/TrpB interface [30]. Residue 125 stabilises the inactive conformation of the active centre [31]. Residues 4, 101, and 153 are all near the active centre. Residues 4 and 125 are in close contact. The role of residue 92 is unclear.

For TrpB, 5 residues possessed a *conn(k*)-value ≥ 4; *conn*(90) = 8 is the highest value. Residue 90 is near the lysine, which binds PLP and catalyses the reaction. Residue 19 is an element of the TrpA/TrpB interface [30] and is at the bottom of the hydrophobic tunnel. Residue 175 is an element of the hydrophobic tunnel [32] and part of the COMM domain, which is involved in the allosteric communication with TrpA [29]. The role of residues 83 and 121 is unclear.

**Figure 4:**
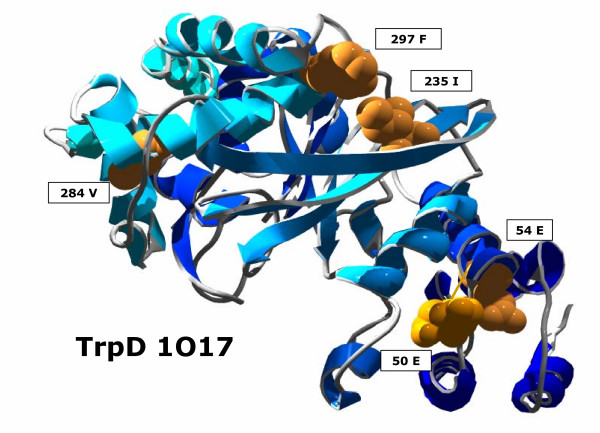
**TrpD residues with highest *conn(k)*-values**. For TrpD (pdb-code 1O17), five residues had *conn(k)*-values ≥ 4. *Conn*(284) was 11.

The anthranilate phosphoribosyl transferase (TrpD) catalyses the group transfer of 5'-phosphoribose from D-5-phosphoribosyl-1-pyrophosphate to the nitrogen atom of anthranilate, which is the third step in L-tryptophan biosynthesis. For TrpD, *H2r *predicted 5 residues as suspicious; see Figure 4. *Conn*(284) was 11. Please note that the residues 235, 297 (*dist*_*min *_= 0.89 Å) and 50, 54 (*dist*_*min *_= 0.72 Å) are contacting residue pairs. For all these residues, the reason for high *conn(k)*-values is unclear.

### H2r as a web-service

We have implemented a server offering *H2r *as a web tool [33]. After uploading a MSA in multiple FASTA format, *H2r *determines bootstrap supported *conn(k)*-values and reports the results *via *email. The web-interface can be utilised to change parameters like the number of *HSRPs *or the usage of pseudo counts. For parameter selection, please see the Additional File 1.

## Discussion

### *conn(k)*-values extend the set of evolutionary relevant residues

Incorporating MSAs turned out to improve the outcome of many applications like *e.g. *the prediction of protein 2D-structure [34] or fold-recognition [35]. The reason is that a MSA describes more precisely the requirements that have to be satisfied at each residue position of a protein. Highly conserved residues tend to correlate with structural or functional importance. Therefore, the identification of conserved residues is *e.g. *relevant for the identification of binding sites [36-38]. A projection of conserved residues onto protein structure helps to identify conservation patterns [39,40]. Correlation analysis as used by *H2r *supplements the repertoire of entropy-based methods of single residues by extending it to residue pairs. The information associated with high *conn(k)*-values is comparable to that of strictly conserved positions: Both signals, which are based on statistical analyses, identify (statistically) suspicious residues. However, in both cases the origin of these signals can only be elucidated by exploiting additional knowledge. A typical example for this enigmatic information is TrpD. 4 of the 5 residues constitute two contacting residue pairs, which supports the significance of the related signal. Nevertheless, the *conn(k)*-values alone do not explain the function of these residues or the origin of the signals.

### *H2r *is a novel approach based on classical, well-proven concepts

Shannon's theory of communication has turned out to be useful in many fields of application. In computational biology, *e.g. *sequence logos are frequently used to assess individual columns in MSAs [18]. A mutual information index *MI*(*k*, *l*) (as defined by formula (4)) was the basis for the work presented in [7]. In biological sequences, *MI *describes the extent of association between residues *k *and *l*.

(4) $$MI(k,l)=\sum_{i,j}f(a_{i}^{k},a_{j}^{l})\log_{2}\frac{f(a_{i}^{k},a_{j}^{l})}{f(a_{i}^{k})f(a_{j}^{l})}\;$$

However, it turned out that unfiltered *MI*-values are a poor indicator for the prediction of co-evolution [8]. Therefore, normalised *MI*-values have been introduced [8].

We prefer *U*(*k*, *l*) as it takes into account the entropy values *H(k) *and *H(l)*, which express the degree of conservation at positions *k *and *l*. *U*(*k*, *l*)-values are normalised and the results deduced from synthetic *MSA_1 *(see Additional File 1) allow us to estimate the coupling strength. Compared to perturbation based methods, *U*(*k*, *l*) has two major advantages: 1) It is less susceptible to signals of a common evolution that might dominate those sequences constituting a perturbation. Generally, these signals are quite strong [41,42]. In addition, we considered this problem by filtering the input and by using bootstrapping. 2) All the information saved in the columns is exploited. The comparison of *U*(*k*, *l*)-values plotted in Additional Figure 2 clearly illustrates the inferiority of the perturbation approach. Comparing *e.g. *the columns representing *frac *values 0.4 and 0.8 illustrates the loss of information. If (say) a perturbation is due to an amino acid occurring in 40% of the sequences, the information content of the remaining 60% of the sequences is ignored. If a second amino acid induces a similarly strong perturbation, the *U*(*k*, *l*)-value increases significantly; compare Additional Figure 2. The same is true for other combinations. A perturbation-based approach does not distinguish between these cases. This example makes clear that the analysis of all frequencies $f(a_{i}^{k},a_{j}^{l})$ significantly strengthens the ability of an algorithm to identify coupled residues.

### Gaps have to be excluded from analysis

**Figure 5:**
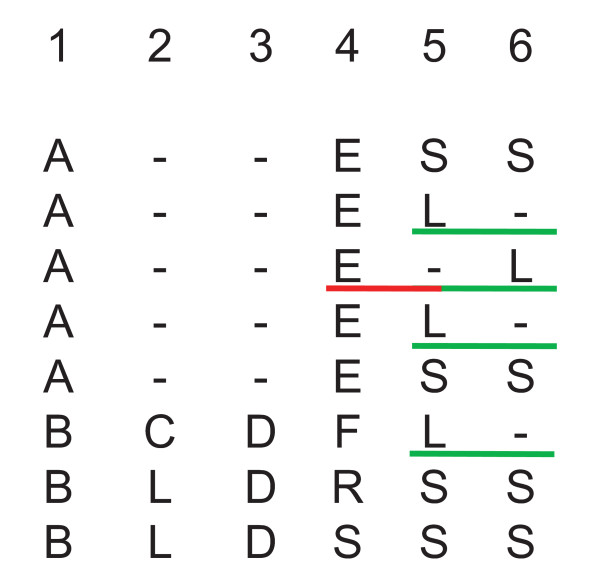
**Modes of handling gaps**. For a correlation analysis, gaps cannot be treated like an additional amino acid. Otherwise *e.g. *column-pairs (1, 2), (1, 3), and (2, 3) would be predicted as possessing a strong coupling signal. Columns 4 – 6 illustrate the computation of frequencies $f(a_{i}^{k},a_{j}^{l})$ for *H2r*. All sequences containing a gap at position *i *or *j *were removed. In the case of columns *i *= 4, *j *= 5 it is one sequence (labelled red). For the determination of frequencies in columns *i *= 5 and *j *= 6, four sequences have to be removed (labelled green).

A well-known problem in the analysis of MSA is the interpretation of columns containing gaps. For the identification of correlation patterns, a gap cannot be treated as 21st amino acid when calculating frequencies. In this case, columns consisting mostly of gaps would be identified as strictly coupled. Figure 5 illustrates the situation: By interpreting gaps as amino acids, column pairs (2, 3) as well as the pairs (1, 2) and (1, 3) would be assigned as being correlated due to the high number of gaps occurring pairwise (signalling a strict coupling) and a certain correlation among the remaining symbols. In addition, positions with a high percentage of gaps would create misleading results for amino acid frequencies due to the small sample size available at those positions. Therefore, it is necessary to eliminate positions containing a certain amount of gaps, as done in [1,24,43-45]. A gap in a sequence means the absence of a residue in the protein structure. Such a deletion is a quite different mutation than a substitution of one amino acid with another one. It follows that insertions and deletions should be treated different from substitutions. However, for the calculation of pseudo counts (see Additional File 1) there exists no model for handling this situation adequately. In addition, one might argue that positions that can be deleted are unlikely to be important for structure or function. These arguments propose to ignore gaps.

On the other hand, ignoring gaps is not appropriate, too. It could be that a substitution of a small side-chain with a large one induces the loss of a residue position. Columns 5 and 6 of Figure 5 illustrate the situation (interpret S as a small and L as a large side chain). Such a correlation cannot be detected when ignoring gaps. In the case of *H2r*, frequencies $f(a_{i}^{k},a_{j}^{l})$ are the basis for computing *U*(*k*, *l*)-values. These frequencies are deduced from those sequences possessing a gap neither at position *k *nor at *l*. Thus, all dependencies, where gaps are not involved, are determined in a correct manner. Therefore, the *U*(*k*, *l*)-values will at all positions solely depend on the signals induced by the amino acid propensities. Thus, ignoring gaps is equivalent to an analysis with 20 instead of 21 symbols. This limitation has to be considered when interpreting *conn(k)*-values.

### *conn(k) *is a robust indicator for co-evolution

**Figure 6:**
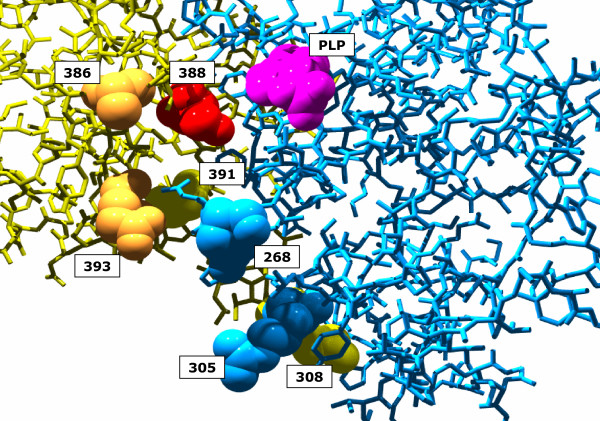
**Location of **1QGN** residues with highest *conn(k)*-values**. The cystathionine *γ *synthase consists of two domains. Residues 48 – 307 constitute the PLP binding domain 1QGN A01 (plotted in blue). Residues 308 – 445 constitute domain 1QGN A02 (plotted in yellow), which binds the substrate cysteine. Residues possessing *conn(k)*-values ≥ 4 were plotted in space filling mode. The *conn(k)*-values were: *conn*(388) = 10, *conn*(268) = 8, *conn*(305) = *conn*(386) = *conn*(391) = *conn*(393) = 5, conn(308) = 4.

Correlation signals can be used to compile networks of residues [12]. In the context of *HSRPs*, simple algorithms are sufficient for cluster and network generation; see Additional File 1. From the analysis of networks, it is known that some nodes may possess a conspicuously high connectivity. Such nodes were named hubs. Hubs hold together large parts of a network. However, what is the meaning of hubs in protein structures? The examples given above may illustrate their role. Cystathionine *γ *synthase (1QGN) consists of two domains [46], which have – according to the CATH database [47] – been designated 1QGNA01 and 1QGNA02. 1QGNA01 binds PLP and consists of residues 48 – 307. 1QGNA02 binds the substrate cysteine [46] and consists of residues 308 – 445. All residues with *conn(k)*-values ≥ 4 are located at the interface of these two domains; compare Figure 6. Residue 388, having the highest *conn(k)*-value of 10, is an element of 1QGNA02, which is not the PLP binding domain. However, this residue is located directly opposite of PLP. For this example, the findings support the notion that *conn(k)*-values identify residues that signal the concerted co-evolution of domains to form a novel protein function. The functional role of residues possessing high *conn(k)*-values in the SH3 domain indicates their importance, too. The same is true for most of the conspicuous TrpA/TrpB residues.

### Future improvements

For a reliable prediction of residues that play a major role in protein function or evolution, robustness has to be implemented on all levels of algorithmic design. For success, the generation of high quality MSAs is a critical step. Both the advent of novel algorithms [48,49] and methods to extract reliable regions [50] plus the wealth of samples originating from completely sequenced genomes put these analyses on a sound basis. Our tests demonstrated that the predictions of *H2r *were not markedly affected by the mode of MSA generation. This indicates that state of the art programmes and datasets like PFAM offer MSAs of similar quality, which proved to be adequate for coupling analysis. Nevertheless, the composition of the samples fed into an algorithm has to be controlled. Assessing the local quality of MSAs as introduced with T-Coffee [51] and the phylogenetic relation of sequences as implemented with CorrMut [26] could be means to further enhance the prediction quality.

In addition, it should be possible to improve the above core algorithm. Shannon's theory does only consider the frequency of symbols and does not regard the features of the represented objects. In the case of MSAs, it would be reasonable to analyse the composition of columns and to assess the properties of occurring amino acids *e.g. *by applying a BLOSUM-like scoring function. This is why we are planning to model the biological context more specifically.

## Conclusion

*conn(k) *is a novel parameter for the characterisation of a specific class of residues. In a robust way, it indicates the strength of co-variation detectable among residues. In contrast to strictly conserved residues, amino acid composition is allowed to vary for these residues. However, the instrumentation of these positions is sensitively balanced with several other ones. Just as strictly conserved residues, these ones offer an enigmatic signal of protein evolution or function. For a complete decoding, knowledge about the protein, its function, and evolution has to be considered.

## Methods

### An entropy based score for the determination of correlations

For a random variable (column) *k*, whose values are linked to a discrete set of frequencies *f(a*_*i*_) of amino acids, the entropy *H *can be computed according to formula (1). The entropy *H(k, l) *of two variables (columns) *k *and *l *is defined by formula (2). In order to measure the dependency of *k *and *l*, the coefficient *U*(*k*, *l*) can be computed according to formula (3). All frequencies have to be deduced from an MSA. An implementation for computing *U*(*k*, *l*) is described in [19].

### Adding pseudo counts

The frequency *f*($a_{i}^{k}$) of each amino acid *a*_*i *_occurring at position *k *was corrected according to [52]:

(5) $$f(a_{i}^{k})=\frac{n(a_{i}^{k})+\lambda \sum_{l=1,l\neq ì}^{20}n(a_{l}^{k})S(a_{i},a_{l})/\sqrt{n(k)}}{n(k)+\lambda \sqrt{n(k)}}$$

*n*($a_{i}^{k}$) is the occurrence of amino acid *a*_*i *_at position *k*, *n*(*k*) is the total occurrence of all amino acids at position *k*, *S*(*a*_*i*_, *a*_*l*_) are Blosum50 [53] scores and *λ *is a weight factor, with 0 ≤ *λ *≤ 1.0. For *H2r*, we used *λ *= 1.0.

The frequencies $f(a_{i}^{k},a_{j}^{l})$ were based on corrected occurrences $n_{corr}(a_{i}^{k},a_{j}^{l})$:

(6) $$n_{corr}(a_{i}^{k},a_{j}^{l})=n(a_{i}^{k},a_{j}^{l})+\lambda \sum_{m=1,m\neq l}^{20}n(a_{i}^{k},a_{j}^{m})S(a_{j},a_{m})/\sqrt{n(k)}$$

$n(a_{i}^{k},a_{j}^{l})$ is the occurrence of pairs of amino acids *a*_*i *_at position *k *and *a*_*j *_at position *l*. *n*(*k*) is the sum of all $n(a_{i}^{k},a_{j}^{l})$ values. The *n*_*corr*_-values were normalised so that the sum of the *n*_*corr*_-values was equal to the sum of the (uncorrected) $n(a_{i}^{k},a_{j}^{l})$ values.

### Assessing residue conservation

For each column of an MSA, the largest frequency of any amino acid *a*_*i *_was determined. If *f*_*max*_(*a*_*i*_) ≥ 0.95, the column and the related residue were regarded as strictly conserved.

### Processing the input

Let *S*_1 _... *S*_*n *_be the *n *sequences constituting the input (MSA) *sequ_in*. For the computation of sequence identity values *ident*, the number of identical residues (ignoring gaps) was determined. The two parameters *ident*_*min *_and *ident*_*max *_defined the minimal and the maximal sequence identity values used for comparison. In pseudo-code the algorithm works as follows:

**Input**: *sequ_in *= {*S*_1_,..., *S*_*n*_}

**Output**: The set *filtered*

Add *S*_1 _to *filtered*

For *i *= 2 *n *do

{

   Compare *S*_*i *_to all sequences of *filtered *and determine *ident*

   If *ident*_*min *_≤ *ident *≥ *ident*_*max *_for all comparisons

      Add *S*_*i *_to *sequ_in*

}

Due to the results of parameter optimisation (see Additional File 1), the default for *ident*_*min *_was 20% and for *ident*_*max *_it was 90%. Columns possessing more than 25% gaps were masked and not processed further. Please note that the first sequence of the input is always an element of the set *filtered*.

### Measuring distances between residues

For measuring distances of residues, we used routines compiled by M. Gerstein [54]. We defined the distance *dist*_*min*_(*k*, *l*) of two residues *k*, *l *as the minimal space between van der Waals radii of any pair of atoms belonging to *k *or *l*, respectively. Thus, a distance of 0 Å indicates that at least two atoms of *k *and *l *are in direct contact in 3D-space.

## Authors' contributions

MZ prepared datasets and multiple sequence alignments and assisted in manuscript writing. RM designed and implemented the algorithm and wrote the manuscript. Both authors read and approved the final version.

## Supplementary Material

Additional File 1 Parameter optimisation and performance tests for *H2r*. Computations used for parameter optimisation and additional performance tests. Click here for file
