# Supplementary material for: H2r: Identification of evolutionary important residues by means of an entropy based analysis of multiple sequence alignments
Source: BMC Bioinformatics. 2008 Mar 18;9:151. doi: 10.1186/1471-2105-9-151 (PMC2323388; doi:10.1186/1471-2105-9-151)
Supplement: Additional File 1 — Parameter optimisation and performance tests for H2r. Computations used for parameter optimisation and additional performance tests. [file 1471-2105-9-151-S1.pdf]

Parameter optimisation and performance tests for *H2r*.

U(k, l) may be used to quantify the specificity of correlated mutations

It has been shown that the outcome of methods exploiting correlated mutations differs quite significantly. The parameter *MI* and effects of normalisation have been characterised exhaustively [1]. In order to access the score *U(k, l)*, which we propose here, we started by analysing synthetic MSAs.

What distinguishes in a MSA two strictly conserved residues from two residues featuring correlated mutations? In both cases, the occurrence of amino acids at two positions *k* and *l* is non-random. At strictly conserved positions, *a specific amino acid* a_i occurs at position *k* and *a specific amino acid* a_j is at position *l*. For correlated mutations, *a specific pattern of amino acid usage* at position *l* is expected for each amino acid a_i at position *k*. In the simplest case, more than one pair of amino acids (a_i, a_j) occurs at residue pairs *k, l*, if these residues mutate in a correlated manner. To characterise *U(k, l)* for such cases, we compiled the synthetic *MSA_1*. It contained 50 columns and 500 sequences (lines). Each column consisted of a certain amount *frac* of conserved residues. The remaining fraction (1.0 - *frac)* of lines was filled with randomly chosen symbols. The 50 columns were organised in 5 blocks. Within each block, the *frac* values 0.2 (relative columns 1, 2), 0.4 (columns 3, 4), 0.6 (columns 5, 6), 0.8 (columns 7, 8), and 1.0 (columns 9, 10) were used. The number of amino acids contributing to the composition of the *frac* part varied. For the first block, one symbol was utilised to fill the *frac* lines of each column. For the second block, two different symbols were selected for each column; for block 5, five symbols contributed with equal amounts to the *frac* lines. Thus, columns 49 and 50, which represent a *frac* value of 1.0, were filled completely with non-randomly chosen symbol. As these two columns belong to block 5, five pairs of symbols contributed with 20%, respectively. All other columns were treated accordingly. Additional Figure 1 illustrates the composition of *MSA_1*.

H2r was used to analyse *MSA_1*. As *H2r* compares each column *k* to each other column *l*, the behaviour of *U(k, l)* could be studied extensively. Those columns were excluded, where an amino acid occurred with a frequency of at least 95%. Columns, distinguished by $f_{max}(a_i)$ ≥ 0.95 were regarded as strictly conserved and were not further processed by *H2r*. We use the term "strictly conserved residue" according to the above definition.

In Additional Figure 2, elements of five groups of columns were compared on their *U(k, l)*-values. The groups subsumed columns with *frac* values of 0.2, 0.4, 0.6, 0.8, and 1.0, respectively. In each group, blocks 2 to 5 were represented, which differ in the number of symbols constituting the *frac* part. As *H2r* excludes strictly conserved residues from further analysis, entries of block 1 were missing in group 5. As expected, the *U(k, l)*-values increased with *frac*. This increase did not markedly depend on the number of symbols constituting the correlated pairs. If two or more symbol-pairs comprised the same amount of conservation, the *U(k, l)*-values were somewhat larger that those of the one-symbol case. Please note that this score does not favour a certain degree of intermediate conservedness as other algorithms do; see [2].

Additional Figure 3 is a plot of the *U(17, l)*-values. In column 17, two symbols constitute a frequency of 40%, each; the remaining 20% of symbols were chosen randomly. The bars show that those columns having a composition, which were in register with column 17, gained high *U(k, l)*-scores, irrespective of the number of symbols filling the columns. Therefore $U(17, 18) > U(17, 49) = U(17, 50) > U(17, 37) = U(17, 38) > U(17, 27)$. In summary, the gradation of the values was as expected.

In Additional Figure 4, all *U(k, l)*-values originating from *MSA_1* were plotted colour-coded. Columns 9 and 10, which represent strictly conserved residues (compare Additional Figure 1), were excluded from further analysis. As already seen, the *U(k, l)*-values did not markedly depend on the number of amino acids that contribute to a certain level of conservation. All values confirmed the stability of the *U(k, l)* approach. *MSA_1* does model some special cases of correlated amino acid frequencies. The above findings confirmed that *U(k, l)* quantifies the coupling of residues for these situations according to expectation. Due to the number of cases that had to be considered, an exhaustive analysis of more complicated combinations was not feasible. Therefore, it is more reasonable to study predictions in the context of protein structure and function, as done here.

High scoring residue pairs form clusters

It is known for some algorithms of correlation analysis that residues predicted to interact are far apart in the protein structure. Additionally, it has been deduced that coupled residues can be classified into two types according to their cluster membership. There exist isolated pairs *k, l* and larger groups of residues which form clusters [3]. For the optimisation of parameters (see below), we focussed on these clusters.

We complemented *H2r* with a module to read pdb-entries, to determine 3D-distances, and to link a protein structure to the columns of an MSA. Thus, we were able to study predictions with respect to their spatial distance. Please note that we define the distance $dist_{min}(k, l)$ of two residues *k, l* as the minimal space to be measured between van der Waals spheres of any pair of atoms belonging to *k* or *l;* see Methods.

Dekker *et al.* have used the MSA constituting PFAM family PF01053 (Cys_Met_Meta) and the corresponding crystal structure of cystathionine γ synthase (pdb-code 1QGN) to benchmark algorithms that assess perturbations [4]. In order to compare the performance of *H2r* with these results, we analysed this dataset, too. Additional Table 1 lists those 20 residue-pairs having the highest *U(k, l)*-values. The distance distribution of these residue pairs is skewed. A KS-test [5] confirmed that their 3D-distances deviated statistically significantly from the distance distribution deduced from all residue pairs ($p << 0.001$). Interestingly, among the first ten entries the residues of three pairs are more than 10Å apart in 3D-space. Most striking is the pair (86, 388). The distance $dist_{min}$ of these two residues is more than 28Å. Without any doubt, no atomic force field ranges that wide. Therefore, two alternatives most plausibly explain this finding: Either it is an artefact (false positive prediction), or these two residues are part of a network interlinking several residues. Ranganathan and co-workers have postulated the existence of connected pathways among residues and have done mutational studies in the PDZ domain which confirmed their predictions [6]. This finding made the second explanation more plausible.

We used a simple neighbour joining algorithm to deduce clusters from *HSRPs*. Starting from a sorted list (compare Additional Table 1), a first cluster was instantiated consisting of those two residues *k* and *l* possessing the highest *U(k, l)*-value. Then, in descending order, sets of residue pairs *m, n* were allocated according to their *U(m, n)*-values. If *m* or *n* was an element of an existing cluster, the sets were merged; otherwise, an additional cluster {*m, n*} was created. Following the arguments of [4], 75 *HSRPs* were used to create clusters. This choice of a cut-off is somewhat arbitrary but uncritical; see [4]. Finally, a set of clusters *CL_MSA* resulted for each MSA.

A minimal spanning tree allows assessing clusters of coupled residue pairs

A generally accepted method for the characterisation of networks and clusters is a minimal spanning tree [7]. In our case, the sets *CL_MSA* had to be processed. During the construction of spanning trees, strictly conserved residues were accepted as additional nodes, if such a

node interlinked two *HSRPs* of *CL_MSA*. The rationale for this approach was that conserved residues cannot be analysed by our coupling analysis but could be elements of an interaction network.

Additional Figure 5 is a plot of the resulting three trees for Cys_Met_Meta. Please note that these trees contain all residues occurring at least once as an element of the 75 *HSRPs*. It is highly unlikely that all *HSRP* residues can be allocated to a small number of clusters (trees) if the selection were random. With high probability, several links should be missing among this extremely small fraction of all possible residue-pairs. In summary, more that 74 000 residue-pairs constitute the complete dataset, and only a fraction of 75 / 74 000 $\approx 1 \cdot 10^{-3}$ was used to construct spanning trees. Most surprisingly, from the 32 residues contributing to these 75 highest scores, 27 were elements of one tree. Two smaller trees with two (residues 155 and 197) or three nodes (residues 164, 403, 404), respectively, were generated. The direct neighbours of residues 164 and 403 were elements of the dominating tree indicating that just one link was missing for merging these trees. Cys_Met_Meta possesses 35 strictly conserved residues. Only five were involved in the network establishing the dominating spanning tree.

Most strikingly, the distances $dist_{min}$ of the residues constituting the spanning tree were much shorter than those listed in Additional Table 1: Only one distance was larger than 10Å and the mean distance was 2.2Å. This finding and the fact that 27 out of 35 residues were elements of one tree strongly support the hypothesis that coupled residues identified by *H2r* are elements of a tightly interconnected network. In order to confirm the validity of this statement, we randomly allocated *U(k, l)*-values to residue pairs and generated spanning trees in 500 (*H2r_train*) or 1000 (PF01053) individual experiments. We determined the mean distance for each spanning tree, both with and without integrating strictly conserved residues. In all experiments, all clusters had a mean distance > 6Å. This finding and the performance tests introduced below further supported the network hypothesis. For the moment, we can state that the minimum spanning tree and its characteristic parameters are proper concepts for the evaluation of residue networks and the mode of their generation.

Selecting the input for *H2r*

Considering the selection of sequences constituting MSAs for correlation analysis, two quite different approaches have been utilised so far. Frequently, sequences have been accumulated by using PSI-BLAST and species-specific hits exceeding a certain E-value were used for

MSA generation [8], [3]. When developing PSI-BLAST, Altschul and co-workers have argued that a large set of closely related sequences carries little more information than a single member but may induce the outvoting of a small number of divergent sequences [9]. Therefore, a sequences weighting scheme has been implemented for PSI-BLAST. For proteins sharing less than 20% identical residues, it is unclear whether they possess an identical fold [10]. This is why we eliminated unrelated sequences. Considering highly similar sequences, we followed the second of the above lines of arguments. *H2r* uses two parameters $ident_{min}$ and $ident_{max}$, which define the minimal and the maximal sequence identity values. All sequences were compared pairwise and only sequences, which possessed at least $ident_{min}$ and at most $ident_{max}$ residues, were accepted for further analysis. This filtering was initiated with the first entry of the MSA. Due to the results of parameter optimisation (see below), the default for $ident_{min}$ is 20% and for $ident_{max}$ it is 90%.

In the case of *H2r*, strictly conserved positions must be filtered out. For each column *k*, *H2r* determines the $f_{\max}(a_i^k)$ value indicating the highest frequency for any amino acid. Two parameters control the analysis of individual columns. If $f_{\max}(a_i^k) \geq frequ_{cons}$, the column is regarded as strictly conserved. If $f_{\max}(a_i^k) \leq frequ_{max}$, the column is further processed. Per default, $frequ_{cons}$ is 95% and $frequ_{max}$ is 90% (see below). For the search of correlated pairs, a certain variability in the amino acid distribution is required for each residue. $frequ_{max}$ assures that at least 10% of the samples vary for each residue. A high fraction of gaps occurring in a column is an indicator for a possibly low alignment quality. Therefore, columns with more than 25% gaps were masked and not processed further.

A test bed for parameter optimisation

As already pointed out, the construction of a high quality MSA is a crucial prerequisite for the analysis of correlated mutations. In order to eliminate the overrepresentation of closely related sequences and to avoid the inclusion of unrelated sequences, all sequences S_i, S_j are usually compared pairwise to determine the number of identical residues $ident(S_i, S_j)$. If $ident(S_i, S_j)$ exceeds the cut-off $ident_{max}$ or falls below $ident_{min}$, S_i, or S_j is eliminated. Instead of fixing these parameters heuristically, we wanted to train them. Two more parameters had to be optimised in the case of *H2r*: 1) $frequ_{max}$, which is the maximal frequency for the occurrence of an amino acid in any column. *H2r* does not analyse residues where any amino

acid occurs with $f(a_i) > frequ_{max}$. 2) λ, the weight factor for adding pseudo counts (see Methods).

In addition, we wanted to test whether the choice of the programme used for the generation of MSAs influences the results. In order to compose a training set, we randomly selected entries of a database containing the names of structures (pdb-files) resolved with high quality (see compilations on web site of [11]). We accepted the first 20 hits that belonged to both a PFAM-MSA and an InterPro family with at least 90 entries. We selected a low cut-off value in order to generate datasets allowing to study the impact of pseudo counts on the quality of the output. One PFAM entry had 91 sequences, a second 164, and the remaining ones more than 200. The mean number of sequences was for the PFAM datasets 429 and for the InterPro entries 391. As an alternative to PFAMs, we generated MSAs by using the respective InterPro family as input for MAFFT [12], which we utilised with the refinement method L-INS-i. We named this dataset, whose elements are listed in Additional Table 2, *H2r_train*.

In order to optimise the above parameters, the mean connectivity and the compactness of spanning trees were analysed. We defined compactness as

$$compactness(tree) = \frac{|(k,l)|}{\sum_{(k,l) \in tree} dist(k,l)} \qquad (6)$$

$|(k, l)|$ is the number of residue pairs contributing to the spanning tree, the denominator adds up the length of all vertices. For each MSA, we analysed the largest spanning tree. Additional Table 3 lists the results. For the set of PFAM MSAs, $ident_{min}$ = 20%, $ident_{max}$ = 90%, and $frequ_{max}$ = 90% gave the best results, both without and after adding pseudo counts (λ = 1.0). For *H2r_train*, we were not able to outperform the PFAM results by creating individual MSAs. Interestingly, adding pseudo counts increased the mean connectivity to a certain degree in both cases.

To study in more detail how the mode of MSA generation influences the results of *H2r*, we focussed on PF01053, which has been analysed previously [4]. We created MSAs by applying several methods to the sequences of IPR000277, which is the related InterPro family [13]. In addition to the above output parameters, we analysed the mean distance between residues of the spanning tree. The results are listed in Additional Table 4. In

summary, the mean distances $dist_{min}$ determined for the spanning trees varied between 1.64Å and 2.96Å. This finding corroborates the above finding that *HSRPs* are tightly linked and form connected networks. This notion is further supported by a mean connectivity of approximately 2.

In order to evaluate the power of an algorithm for predicting physically close residues, the 3D-distances of 75 non-trivial high scoring pairs have been analysed [4]. Non-trivial residue pairs are those, which are more than 8 residues apart in the primary sequence. These distances were compared to the median distance deduced from all residue pairs of the dataset. The quotient of the fractions of *HSRPs* lying below or above the median has been proposed as a quality criterion [4]. For PF01053, Ranganathan's approach reached 56 / 19, whereas the ELSC algorithm gained 62 / 13; see [4]. Additional Table 4 affirms that *H2r* used with default parameters outperforms both approaches. The data also confirm that the specific choice of the parameters and the mode of generating MSAs have only a minor effect on *H2r*'s results.

The pre-processed MSAs accommodate a stable signal

Because of our rigorous filtering procedure, the MSAs extracted from a full PFAM alignment were relatively small. For example, the full PFAM0067 contained 8703 sequences. After filtering, the resulting MSA consisted of 534 entries. In order to test the robustness of *H2r* with regard to the number of sequences constituting the input, we filtered with default parameters all PFAMs belonging to the dataset *H2r_train* and selected those 10 MSAs consisting of more than 150 sequences; Additional Table 5 lists relevant parameters. We named this set of MSAs *H2r_filt_100*. For every MSA(*i)* ∊ *H2r_filt_100*, we created 25 MSAs consisting each of a randomly selected fraction of 75%, and 25 MSAs consisting each of a randomly selected fraction of 60% of the sequences belonging to MSA(*i)*. We named these samples *H2r_filt_75* and *H2r_filt_60.* For each of these 500 MSAs, we determined *conn(k)*-values and compared the outcome to that of *H2r_filt_100.* We named a residue *k* possessing a *conn(k)*-value above the MSA-specific threshold a *distinguished* residue. We used *Dist_100* to name the set of residues being *distinguished* in *H2r_filt_100;* accordingly, we used the names *Dist_75* and *Dist_60*. *Dist_75* consists of those residues being *distinguished* in at least one of the 250 MSAs ∊ *H2r_filt_75.*

For the 10 MSAs of *H2r_filt_100*, *H2r* identified 50 *distinguished* residues; see Additional Table 5. The distribution of *conn(k)*-values was 4 (*conn(k)* = 4), 20 (*conn(k)* = 5), 8 (*conn(k)*

= 6), 3 (*conn(k)* = 7), 2 (*conn(k)* = 8) and 13 (*conn(k)* ≥ 9). We determined how frequently these residues of *Dist_100* were also *distinguished* in all of the MSAs of *H2r_filt_75* and *H2r_filt_60*. For the 75% samples the correspondences were 50% (*conn(k)* = 4 or 5), 84% (*conn(k)* = 6), 95% (*conn(k)* = 7), 96% (*conn(k)* = 8) and 99% (*conn(k)* ≥ 9). For the 60% samples these rates were 48% (*conn(k)* = 4 or 5), 78% (*conn(k)* = 6), 87% (*conn(k)* = 7), 92% (*conn(k)* = 8) and 93% (*conn(k)* ≥ 9). A correspondence of 84% means that all of the respective residues had a *conn(k)*-value above the MSA-specific cut-off in 21 out of the 25 MSAs constituting *H2r_filt_75*. Please note that all residues possessing a *conn(k)*-value ≥ 6 in *H2r_filt_100* were *distinguished* in at least 21 of the 25 datasets constituting *H2r_filt_75.* For *H2r_filt_60* these were 20 out of 25 datasets, respectively.

In addition, we characterised the set of residues being not an element of *Dist_100* but belonging to *Dist_75* or *Dist_60,* respectively. For the 75% samples, these were 12 residues; for the 60% samples, these were 33. As some residues were *distinguished* in several of the 25 analyses, 49 times a *conn(k)*-value above the threshold occurred for the 75% samples. The occurrences of assigned *conn(k)*-values were 39 (*conn(k)* = 4 or 5), 9 (*conn(k)* = 6), and 1 (*conn(k)* = 7 or 8) for the 75% samples and 63 (*conn(k)* = 4 or 5), 13 (*conn(k)* = 6), and 16 (*conn(k)* = 7 or 8) for the 60% samples. This means that altogether a *conn(k)*-value ≥ 6 was assigned 10 times to a residues not *distinguished* in *H2r_filt_100* in all of the 250 MSAs of *H2r_filt_75*. For the 60% samples, these were 29 cases. Additional Table 5 list the occurrence of these additionally *distinguished* residues. For the 60% samples, 18 of these *distinguished* residues occur in PFAM00068 and PF00557. The corresponding MSAs of the 60% samples contained 137 or 113 sequences, respectively. As explained above, approximately 125 sequences are a lower limit for the determination of *U(k, l)* values [1]. Therefore, the small sample size most plausibly explains the higher variation of *distinguished* residues in *H2r_filt_60*. When assessing the number of additional residues possessing *conn(k)*-values of 4 or 5 in the 75% and 60% datasets one has to consider that 20 residues of *H2r_filt_100* had a *conn(k)*-value of 3, which was too low for a "distinguishment" in *H2r_filt_100*. Thus, relatively small fluctuations in the correlation signals made these residues to *distinguished* ones in *H2r_filt_75* or *H2r_filt_60*.

In order to study these fluctuations in more detail, we used the above results for a bootstrap analysis. For each element of *Dist_75* and *Dist_60*, we determined its mean *conn(k)*-value and a bootstrap-value. Here, the bootstrap-value was the number of analyses, where *conn(k)*

exceeded the MSA-specific cut-off. A bootstrap of 80% indicates that *k* was *distinguished* in 20 out of the 25 samples. Additional Figure 6 presents the results. The plots show a clear correlation of mean *conn(k)*-values and those determined for *H2r_filt_100*. A Spearman rank correlation of the results for *H2r_filt_100* with that for *H2r_filt_75* and *H2r_filt_60* gave clear correlations with $r = 0.90$ ($p << 0.01$) and $r = 0.88$ ($p << 0.01$).

Only 4 predictions of *Dist_75,* which did not belong to *Dist_100* had a mean *conn(k)*-value > 4. Only 4 predictions of *Dist_100* had a mean *conn(k)*-value ≤ 4 in the 75% samples, for the 60% samples these were 7. The bootstrap-values further confirmed the robustness of the *H2r* approach: Only 3 of the predictions belonging to *Dist_75* but not to *Dist_100* possessed a *conn(k)*-value > 4 and were supported by a bootstrap value ≥ 50%. These residues had a *conn(k)*-value of 4 in *H2r_filt_100*, which was below the cut-off for a "distinguishment" in *H2r_filt_100*. For *Dist_60* one additional residue was *distinguished*. In summary, these analyses showed that the outcome of *H2r* does not markedly depend on the specific sample representing a protein family, if the input has been filtered rigorously.

Selecting 75 *HSRPs* is a reasonably starting point

The number of highest scoring residue pairs *(HSRPs)* used to deduce *conn(k)*-values is one of the parameters of *H2r*. Following previously introduced arguments [4], we analysed 75 *HSRPs* for each experiment. In order to demonstrate that this cut-off is no critical parameter, we assessed PF01053 by using 50, 75, 100, 150, 375, and 750 *HSRPs*. For this MSA, 75 *HSRPs* are approximately 1‰ of all residue pairs. Additional Table 6 summarises the resulting *conn(k)*-values and their ranks. Due to the differing number of *HSRPs*, the magnitude of *conn(k)*-values varied. However, the ranking of residues on their *conn(k)*-values was highly correlated. A rank correlation [14] of *conn(k)*-values resulting from 75 *HSRPs* with the results for 50, 100, and 150 *HSRP* values gave a statistically significant correlation ($p \leq 0.01$) in each case. These findings indicate that the order of *conn(k)*-values does not critically depend on this parameter. Taking a too large number of *HSRPs* introduced noise: This is the case for results listed in the last two columns of Additional Table 6, which are due to an evaluation of approximately 5‰ or 10‰ of *U(k, l)*-values.

One might argue that the number of *HSRPs* selected for the determination of *conn(k)*-values should be chosen depending on the length of the sequences constituting the MSA. In order to assess this parameter, we used the sequences of *H2r_filt_100* and selected 50, 75 and 150 *HSRPs* as well as 1‰, 2.5‰, and 5‰ of sequence length. Additional Table 5 lists the

results. In summary, this setting did not markedly influence the identification of those residues having highest *conn(k)*-values. The comparison of the *HSRPs* = 75 and the 2.5‰ results showed that the outcome overlapped to a great extent. For short sequences, a larger percentage of residues has to be chosen. There was no obvious trend in the dependency of the rank of *conn(k)*-values and the number of *HSRPs* used for computation. Therefore, we propose as a starting point *HSRPs* = 75. However, the web server [15] accepts different values.

Mode of MSA generation is uncritical

In order to characterise *conn(k)*-values with respect to the mode of MSA generation, we created additional alignments. Several input filters and methods of MSA generation were applied to the sequences of PF01053 and IPR000277, which is the corresponding InterPro family [13]. As MSAs used for coupling analysis contain large numbers of sequences, we focussed on MAFFT [12], which combines accuracy and speed [16]. Altogether, 11 different MSAs were generated; see Additional Table 4. For each element of a *HSRP*, we recorded the minimal and maximal connectivity value observed in any experiment. Additional Table 7 summarises the results. 43 residues were an element of at least one *HSRP.* For 12 residues, their occurrence showed a skewness that might be caused by a specific dataset or a method of MSA generation. 6 of these residues occurred in PFAM specific datasets, 6 in those MSAs originating from InterPro sequences by using MAFFT. The two refinement modes of MAFFT (FFTNS or L-INS-i), which we tested did not contribute a suspiciously high number of extra residues. Importantly, none of these 12 extra residues gained in any of the experiments a *conn(k)*-value > 3. The set of *HSRPs* originating from a realignment of PF01053 generated by means of MAFFT did not notably deviate from the above set of *HSRPs* (data not shown).

The output of algorithms for correlation analysis differs significantly

In *H2r_filt_100,* each PFAM is associated with a protein-structure being resolved with high quality. In order to characterise a larger set of proteins, *conn(k)*-values were compared with the outcome of CorrMut [17] and P2PConPred [18]. For both programmes, the protein structure representing the PFAM (see Additional Table 8) and default settings were used as input. In the case of CorrMut, the list of residue pairs was sorted on *i*) the Pearson correlation value and *ii*) the expected Pearson correlation value, which are alternative parameters for weighting the strength of the correlation signal. For each protein, 5 residue pairs with lowest

ranks were added to Additional Table 8. In the case of P2PConPred, those 5 residue pairs possessing the highest correlation scores were listed. The comparison of the results showed that the predictions did not correspond to a great extent. Altogether, 4 residues were predicted as being involved in correlations by at least two algorithms.

It is unclear, why the outcome of the programmes is so different: Both CorrMut and P2PConPred use the sequence of the protein given as input as a query for an internal BLAST search. In the case of CorrMut, the analysis is a phylogeny-based reconstruction of mutations. In the case of P2PConPred, substitution matrices are the basis for the computation of correlation scores. Presumably, the algorithms identify different evolutionary signals. In summary, these results confirm that *H2r* identifies a specific subset of residues, which does only marginally overlap with the outcome of other algorithms for correlation analysis.

References

1. Martin LC, Gloor GB, Dunn SD, Wahl LM: **Using information theory to search for co-evolving residues in proteins.** *Bioinformatics* 2005, **21:**4116-4124.
2. Fodor AA, Aldrich RW: **Influence of conservation on calculations of amino acid covariance in multiple sequence alignments.** *Proteins* 2004, **56:**211-221.
3. Gloor GB, Martin LC, Wahl LM, Dunn SD: **Mutual information in protein multiple sequence alignments reveals two classes of coevolving positions.** *Biochemistry* 2005, **44:**7156-7165.
4. Dekker JP, Fodor A, Aldrich RW, Yellen G: **A perturbation-based method for calculating explicit likelihood of evolutionary co-variance in multiple sequence alignments.** *Bioinformatics* 2004, **20:**1565-1572.
5. Press WH, Teukolsky SA, Vetterling WT, Flannery BP: *Numerical recipes in C.* Cambridge: Cambridge University Press 1992.
6. Lockless SW, Ranganathan R: **Evolutionarily conserved pathways of energetic connectivity in protein families.** *Science* 1999, **286:**295-299.
7. Kruskal J: **On the shortest spanning subtree of a graph and the traveling salesman problem.** *Proc Amer Math Soc*, **7:**48-50.
8. Süel GM, Lockless SW, Wall MA, Ranganathan R: **Evolutionarily conserved networks of residues mediate allosteric communication in proteins.** *Nat Struct Biol* 2003, **10:**59-69.
9. Altschul SF, Madden TL, Schaffer AA, Zhang J, Zhang Z, Miller W, Lipman DJ: **Gapped BLAST and PSI-BLAST: a new generation of protein database search programs.** *Nucleic Acids Res* 1997, **25:**3389-3402.
10. Sander C, Schneider R: **Database of homology-derived protein structures and the structural meaning of sequence alignment.** *Proteins* 1991, **9:**56-68.
11. Vriend G: **WHAT IF: a molecular modeling and drug design program.** *J Mol Graph* 1990, **8:**52-56.
12. Katoh K, Kuma K, Toh H, Miyata T: **MAFFT version 5: improvement in accuracy of multiple sequence alignment.** *Nucleic Acids Res* 2005, **33:**511-518.
13. Mulder NJ, Apweiler R, Attwood TK, Bairoch A, Bateman A, Binns D, Bork P, Buillard V, Cerutti L, Copley R, et al: **New developments in the InterPro database.** *Nucleic Acids Res* 2007, **35:**D224-228.
14. Spearman C: **The proof and measurement of association between two things.** *Am J Psychol* 1904, **15:**72-101.
15. H2r-online [http://www-bioinf.uni-regensburg.de].
16. Nuin PA, Wang Z, Tillier ER: **The accuracy of several multiple sequence alignment programs for proteins.** *BMC Bioinformatics* 2006, **7:**471.
17. Fleishman SJ, Yifrach O, Ben-Tal N: **An evolutionarily conserved network of amino acids mediates gating in voltage-dependent potassium channels.** *J Mol Biol* 2004, **340:**307-318.
18. Eyal E, Frenkel-Morgenstern M, Sobolev V, Pietrokovski S: **A pair-to-pair amino acids substitution matrix and its applications for protein structure prediction.** *Proteins* 2007, **67:**142-153.

Additional Figures

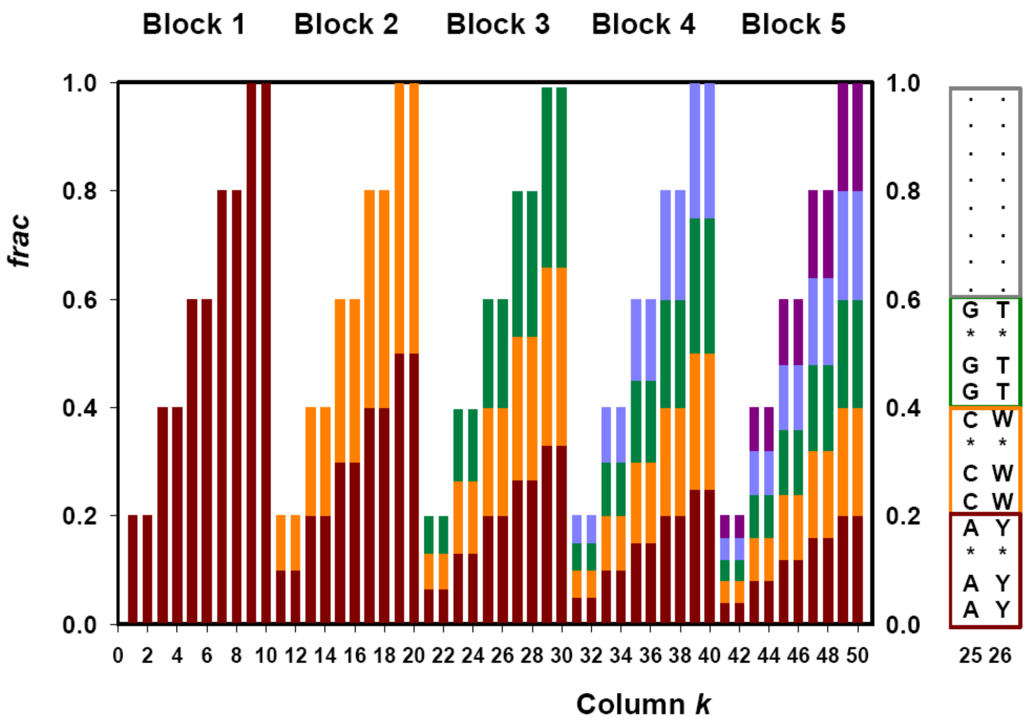

Additional Figure 1 - Composition of synthetic *MSA_1*

An MSA consisting of 50 columns and 500 lines (sequences) was generated. Ten columns each constituted a block. Within each block, columns were organised in pairs. For each pair, a fraction of *frac* lines was filled with identical symbols; the remaining fraction (1.0 – *frac*) of lines was filled with randomly chosen symbols. For *frac*, the values 0.2, 0.4, 0.6, 0.8, and 1.0 were utilised. The number of symbols used to fill the *frac* lines increased from 1 in block 1 to 5 in block 5. The schema given on the right illustrates the composition of columns 25 and 26. According to the *frac* value of 0.6, the lower 60% of the columns were filled with non-randomly chosen symbols. As these two columns belong to block 3, three symbols each were used. In column 25, the symbols A, C, and G occurred in 20% of the sequences, respectively. In column 26, the symbols Y, W, and T were used. Thus, three symbol-pairs (A-Y), (C-W), and (G-T) contributed equally to the constant part.

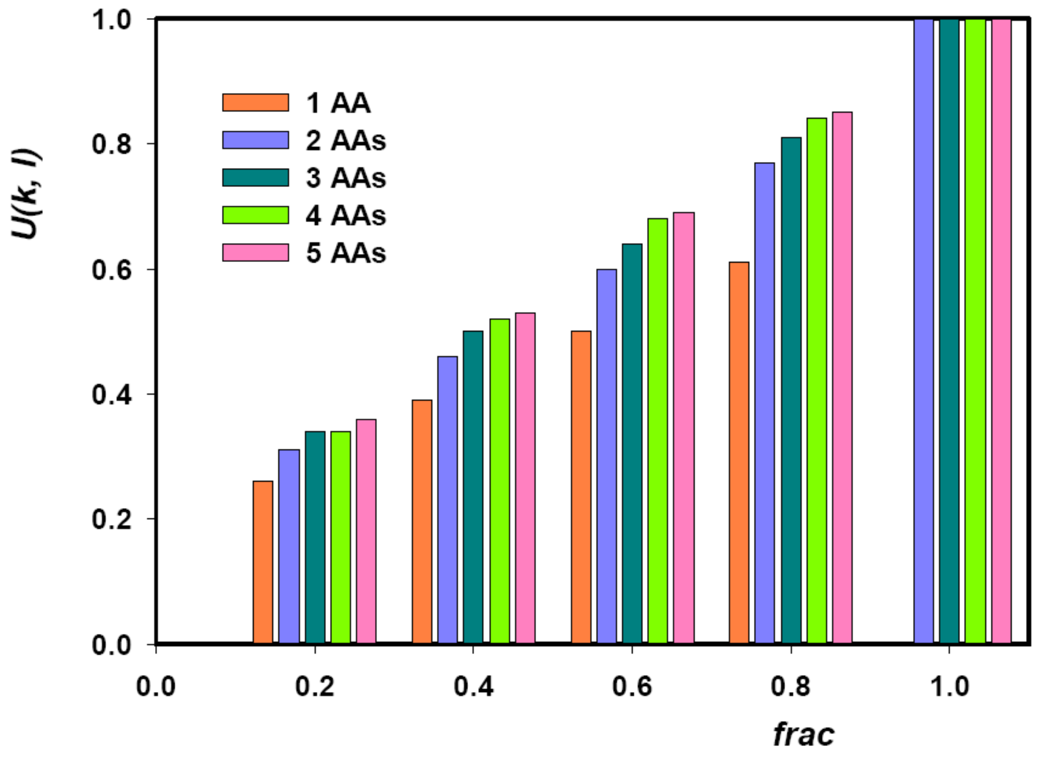

Additional Figure 2 - *U(k, l)*-values *versus* conservation and the number of coupled residue-pairs

Column-pairs originating from *MSA_1* were grouped according to their *frac* value and *U(k, l)*-values were plotted. Each group - but not the last one - contains columns belonging to block 1 – block 5. Elements from various blocks differ in the number of symbols (AAs) constituting the *frac* part. The middle bar of the *frac* 0.6 block originates from the analysis of columns 25 and 26 (compare Additional Figure 1). As $f_{max}(a) > 95\%$ for columns 9 and 10, elements of block 1 are missing in the last group.

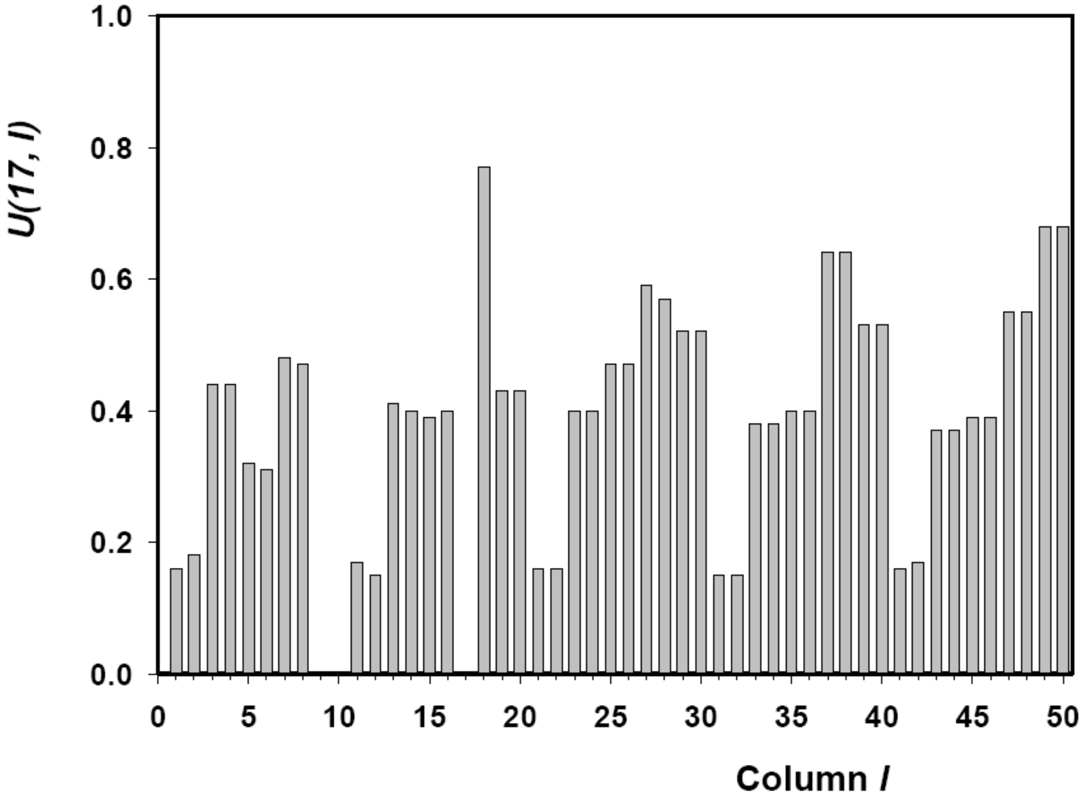

Additional Figure 3 - *U*(17, *l*)-values for all columns *l* of *MSA_1*

All scores resulting from the comparison of column 17 of *MSA_1* to all other columns were plotted.

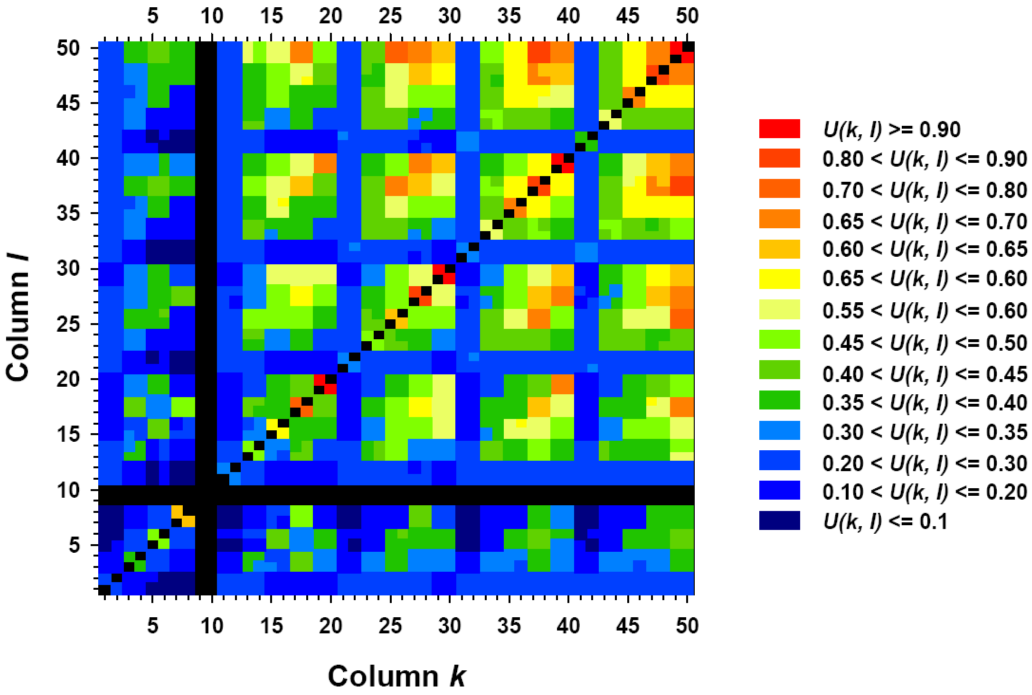

Additional Figure 4 - Colour-coded plot of all *U(k, l)*-values originating from the analysis of *MSA_1*

H2r was used to analyse *MSA_1*. The resulting *U(k, l)*-values were colour-coded according to the scheme given on the right. Columns 9 and 10 mimic strictly conserved residues and were excluded from further analysis.

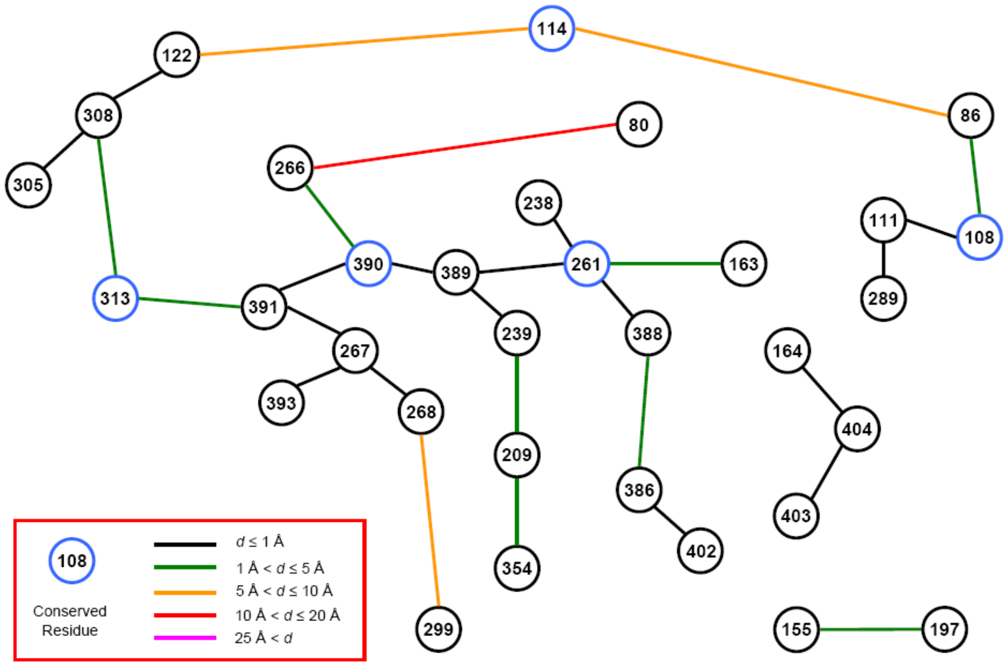

Additional Figure 5 - Minimal spanning trees deduced from most extreme *U(k, l)*-values of PF01053

H2r was used to analyse the MSA of PF01053 (Cys_Met_Meta). Three minimal spanning trees resulted, when clustering 75 residue-pairs possessing highest *U(k, l)*-values. A strictly conserved residue *m* was added to a tree, if *m* linked two of the high-scoring residues. The 3D-distance $dist_{min}$ of pairs *k, l* is colour-coded. Location of nodes and edge lengths are arbitrary and do not reflect 3D-information. Please note that we determined distances $dist_{min}(k, l)$ as the minimal distance of van der Waals surfaces, see Methods.

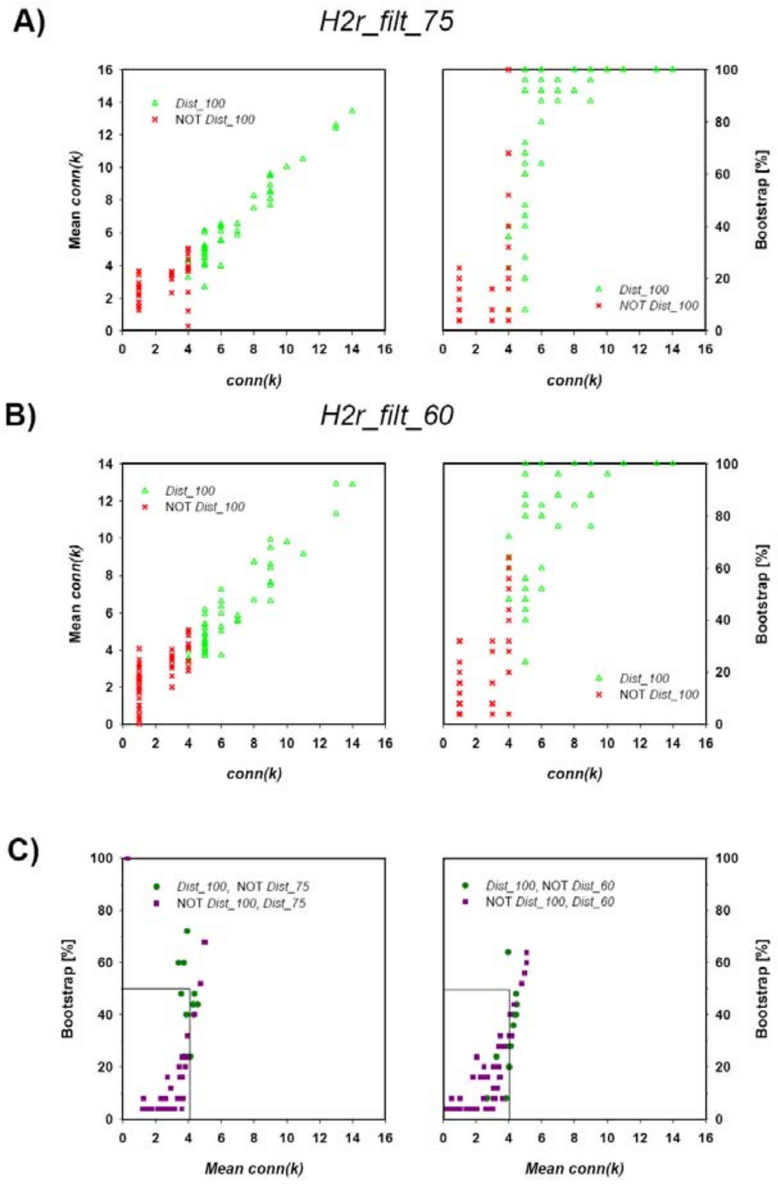

Additional Figure 6 - Effects of sampling on the outcome of *H2r*

For each of the 10 MSAs of *H2r_filt_100*, 25 MSAs were generated by randomly choosing 75% or 60% of the sequences. These two sets of MSAs were named *H2r_filt_75* or *H2r_filt_60*. *Dist_100* are those residues having assigned a *conn(k)*-value above the cut-off in *H2r_filt_100*. NOT *Dist_100* are those residues having no *conn(k)*-value above the cut-off in *H2r_filt_100* but were *distinguished* in at least one of the 250 MSAs of *H2r_filt_75 (*or *H2r_filt_60)*. *Dist_100*, *NOT Dist_75* are those residues having a *conn(k)*-value above the cut-off in *H2r_filt_100* but not in *H2r_filt_75*. All other abbreviations were used accordingly. Mean *conn(k)*-values resulted from the 25 MSAs constituting *H2r_filt_75* or *H2r_filt_60*. Bootstrap-values give for each residue the fraction of *conn(k)*-values above the cut-off in all of the 25 samples. Panel A lists the results for comparing the outcome of *H2r_filt_100* and *H2r_filt_75*. Panel B *ditto* for *H2r_filt_60*. In Panel C, mean *conn(k)*- and bootstrap-values were plotted for those residues being *distinguished* in one dataset but not in the other one.

Additional Tables

Additional Table 1 - 20 high scoring residue pairs of PF01053 as identified by *H2r*

#	Pos *k*	a^k	$f_{max}(a^k)$	Pos *l*	a^l	*U(k, l)*	$dist_{min}$ [Å]
1	305	R	0.57	268	D	0.73	4.75
2	305	R	0.57	388	S	0.58	14.43
3	268	D	0.58	388	S	0.58	6.00
4	267	N	0.47	391	G	0.56	0.85
5	388	S	0.56	393	E	0.56	6.66
6	305	R	0.57	386	A	0.53	18.75
7	386	A	0.46	268	D	0.52	9.36
8	86	A	0.49	388	S	0.51	28.35
9	386	A	0.46	389	F	0.50	5.86
10	389	F	0.56	239	F	0.49	0.62
11	393	E	0.49	308	K	0.49	12.94
12	163	Y	0.87	238	T	0.49	4.90
13	266	H	0.85	238	T	0.49	10.63
14	308	K	0.43	388	S	0.48	16.09
15	391	G	0.47	388	S	0.48	4.75
16	268	D	0.58	393	E	0.47	4.60
17	388	S	0.56	402	M	0.47	5.29
18	391	G	0.47	393	E	0.46	2.13
19	403	S	0.76	404	Y	0.46	0.98
20	393	E	0.49	305	R	0.45	8.62

The first column lists the rank deduced from the *U(k, l)*-values. Pos *k* and *l* give the number of the residues; a^k and a^l are the amino acids occurring at these positions in pdb-entry 1QGN. $f_{max}(a^k)$ is the maximal frequency of any amino acid occurring in column *k*. *U(k, l)* is the score calculated according to formula (3). "$dist_{min}$" gives the distance $dist_{min}$ of the residues in Angström; for calculation see Methods. These values were computed without adding pseudo counts.

Additional Table 2 - Test set *H2r_train* used for parameter optimisation

PDB	PFAM	InterPro
1WKR	PF00026	IPR001461
1JFB	PF00067	IPR001128
1G4I	PF00068	IPR001211
1P7W	PF00082	IPR000209
1F94	PF00087	IPR003571
1HJ9	PF00089	IPR001254
1PA2	PF00141	IPR002016
1GVE	PF00248	IPR001395
1R85	PF00331	IPR001000
1I76	PF00413	IPR001818
1KQR	PF00426	IPR000416
1KV0	PF00537	IPR002061
1T8K	PF00550	IPR006163
1QXY	PF00557	IPR000994
1G6I	PF01532	IPR001382
1UWC	PF01764	IPR002921
1GK9	PF01804	IPR002692
1VMH	PF01894	IPR001602
1G61	PF01912	IPR002769
1JU2	PF05199	IPR007867

The dataset comprises 20 combinations of 3D-structures resolved with high quality and the related PFAM families and InterPro datasets. For structures, their pdb-code is given.

Additional Table 3 - Mean connectivity and compactness values for *H2r_train* resulting from training

$frequ_{max}$	Sequence Identity [%]																			
	0-80	0-90	0-95	0-99	0-100	10-80	10-90	10-95	10-99	10-100	20-80	20-90	20–90<br>PsC	20-95	20-100	25-80	25-90	25-95	25-99	25-100
**0.75**	1.79<br>1.59<br>0.56<br>0.46	1.77<br>1.65<br>0.52<br>0.46	1.75<br>1.62<br>0.55<br>0.50	1.76<br>1.63<br>0.56<br>0.49	1.77<br>1.64<br>0.58<br>0.47	1.81<br>1.59<br>0.56<br>0.48	1.76<br>1.63<br>0.54<br>0.46	1.76<br>1.57<br>0.55<br>0.52	1.74<br>1.57<br>0.56<br>0.49	1.72<br>1.53<br>0.60<br>0.48	1.60<br>1.45<br>0.61<br>0.48	1.64<br>1.48<br>0.56<br>0.44	1.92<br>1.79<br>0.56<br>0.45	1.65<br>1.46<br>0.61<br>0.47	1.62<br>1.50<br>0.59<br>0.50	1.63<br>1.67<br>0.59<br>0.48	1.70<br>1.71<br>0.58<br>0.51	1.71<br>1.46<br>0.59<br>0.46	1.70<br>1.72<br>0.63<br>0.51	1.67<br>1.70<br>0.66<br>0.52
**0.90**	1.85<br>1.77<br>0.66<br>0.66	1.78<br>1.84<br>0.57<br>0.50	1.78<br>1.84<br>0.57<br>0.52	1.74<br>1.73<br>0.60<br>0.47	1.78<br>1.75<br>0.62<br>0.46	1.75<br>1.65<br>0.55<br>0.49	1.84<br>1.66<br>0.58<br>0.45	1.77<br>1.64<br>0.58<br>0.46	1.73<br>1.54<br>0.58<br>0.49	1.73<br>1.52<br>0.61<br>0.48	1.67<br>1.40<br>0.60<br>0.53	1.85<br>1.47<br>0.56<br>0.49	2.01<br>1.74<br>0.56<br>0.49	1.79<br>1.52<br>0.59<br>0.46	1.74<br>1.46<br>0.59<br>0.49	1.68<br>1.50<br>0.57<br>0.51	1.85<br>1.73<br>0.54<br>0.52	1.77<br>1.52<br>0.59<br>0.48	1.72<br>1.71<br>0.62<br>0.52	1.68<br>1.69<br>0.61<br>0.52
**0.95**	1.83<br>1.76<br>0.65<br>0.66	1.86<br>1.82<br>0.58<br>0.58	1.74<br>1.81<br>0.57<br>0.51	1.74<br>1.78<br>0.57<br>0.45	1.78<br>1.73<br>0.59<br>0.46	1.83<br>1.63<br>0.61<br>0.50	1.81<br>1.65<br>0.57<br>0.53	1.83<br>1.61<br>0.59<br>0.45	1.73<br>1.64<br>0.59<br>0.48	1.71<br>1.62<br>0.62<br>0.45	1.76<br>1.39<br>0.59<br>0.52	1.82<br>1.46<br>0.58<br>0.48	1.99<br>1.74<br>0.56<br>0.48	1.86<br>1.48<br>0.60<br>0.52	1.77<br>1.45<br>0.60<br>0.48	1.64<br>1.62<br>0.57<br>0.50	1.80<br>1.82<br>0.54<br>0.52	1.74<br>1.84<br>0.56<br>0.56	1.74<br>1.80<br>0.63<br>0.52	1.70<br>1.81<br>0.63<br>0.52

All elements of the datasets *H2r_train* given in Additional Table 2 were analysed by using *H2r*. For each run, a specific combination of $ident_{min}$, $ident_{max}$, and $frequ_{max}$ values were selected. For each experiment four results are given. The upper two numbers list the mean *conn(k)*-values for the PFAM and the InterPro datasets, the lower two numbers are the mean compactness values. The column labelled PsC lists the results for an experiment using pseudo counts with $\lambda = 1.0$. **Example**: Analysing the PFAM dataset with $ident_{min}$ = 10%, $ident_{max}$ = 90% and $frequ_{max}$ = 0.90 resulted for the PFAM dataset in a mean connectivity of 1.84 and a mean compactness of 0.58. Using the same parameters for the InterPro data gave a connectivity of 1.66 and a compactness of 0.45.

Additional Table 4 - Characterisation of 11 MSAs representing Cys_Met_Meta

#	Dataset	Non-trivial pairs	Mean Connectivity	Compact-ness	Mean Distance [Å]
1	PF01053, 20 – 100, 95	67 / 8	1.60	0.44	2.29
2	PF01053, 0 – 100, 90	63 / 12	1.50	0.61	1.64
3	PF01053, 20 – 90, 80	60 / 15	1.95	0.51	1.95
4	PF01053, 20 – 90, 90	63 / 12	1.89	0.46	2.19
5	IPR000277, 20 – 90, 90, FFTNS	64 / 11	1.57	0.41	2.44
6	IPR000277, 20 – 90, 90, FFTNS LF	61 / 14	1.83	0.38	2.60
7	IPR000277, 10 – 90, 90, FFTNS	65 / 10	1.50	0.48	2.07
8	IPR000277, 25 – 90, 95, FFTNS LF	59 / 16	2.14	0.41	2.46
9	IPR000277, 20 – 90, 90, LINSI	59 / 16	1.67	0.37	2.67
10	IPR000277, 20 – 90, 90, LINSI LF	63 / 12	1.71	0.34	2.96
11	IPR000277, 10 – 100, 90, LINSI LF	68 / 7	1.64	0.58	1.73

4 different parameter sets were used to analyse PFAM entry PF01053 (lines 1 - 4). 7 different combinations of MSA compilations and parameter sets were utilised for the InterPro entry IPR000277, which corresponds to PF01053. Each dataset is labelled with name, $ident_{min}$ – $ident_{max}$, $frequ_{max}$, [mode of generation]. Name indicates the origin of the sequences, $ident_{min}$ and $ident_{max}$ are the cut-off values for filtering, $frequ_{max}$ is the maximal frequency of any amino acid, mode of generation gives the MAFFT options, LF indicates that the length of the sequences was used for filtering, too. The column "non-trivial pairs" gives the distribution of 75 non-trivial, high scoring residue pairs below and above the median distance of all residue pairs. "Mean Connectivity" is the mean connectivity value. Compactness is the ratio |residues| / Σ distances. "Mean Distance" gives the mean of all $dist_{min}$ values deduced from all pairs constituting the largest spanning trees, respectively.

Additional Table 5 - Resulting *conn(k)*-values for the dataset *H2r_filt*

**PFAM**<br>**PDB, chain,**<br>**Length of MSA**<br>**max *U(k,l)* val**	**Sequences**<br>**PFAM**<br>**Filt**	***HSRPs***						**Add.**<br>**residues**<br>***H2r_filt_75***<br>***H2r_filt_60***
		**50**	**75**	**150**	**1‰**	**2.5‰**	**5‰**	
PF000026<br>1WKR, A<br>338<br>0.40	1337<br>341	291 (5)<br>[209] (3)<br>322 (3)	**236** (6)<br>**[209]** (5)<br>**291** (5)<br>*91 (3)*<br>*205 (3)*<br>*322 (3)*	236 (13)<br>[209] (10)<br>291 (6)<br>91 (5)<br>98 (5)<br>322 (5)	236 (11)<br>[209] (8)<br>291 (6)<br>[39] (4)<br>91 (4)	**[209]** (23)<br>**236** (22)<br>**291** (11)<br>98 (9)<br>238 (9)	236 (38)<br>[209] (33)<br>238 (20)<br>286 (18)<br>291 (15)<br>98 (12)<br>205 (12)	2<br>2
PF000067<br>1JFB, A<br>397<br>0.25	8703<br>534	283 (8)<br>287 (6)<br>291 (6)<br>289 (5)<br>290 (4)	**283** (9)<br>**291** (9)<br>**287** (8)<br>**289** (5)<br>**314** (5)<br>*310 (4)*	283 (17)<br>291 (14)<br>287 (12)<br>310 (11)	283 (17)<br>291 (15)<br>287 (13)<br>310 (11)<br>289 (10)	**283** (32)<br>**291** (28)<br>**287** (21)<br>**289** (21)<br>310 (20)<br>**314** (18)<br>351 (18)	283 (44)<br>291 (42)<br>290 (35)<br>287 (33)<br>289 (32)<br>310 (31)	1<br>0
PF00068<br>1G4I, A<br>123<br>0.48	676<br>229	41 (7)<br>110 (5)<br>11 (4)<br>50 (4)<br>77 (4)<br>14 (3)<br>55 (3)<br>115 (3)	**41** (9)<br>**11** (6)<br>110 (6)<br>**50** (5)<br>**77** (5)<br>85 (5)<br>115 (5)<br>*14 (3)*	41 (13)<br>110 (13)<br>11 (11)<br>77 (11)<br>14 (8)<br>46 (8)<br>50 (8)<br>115 (8)	41 (3)	**41** (5)<br>**50** (4)<br>**77** (4)<br>**11** (3)<br>14 (3)	41 (8)<br>11 (6)<br>50 (6)<br>77 (6)<br>110 (5)<br>115 (5)	4<br>6
PF000089<br>1HJ9, A<br>217<br>0.38	6237<br>345	152 (9)<br>101 (5)<br>27 (49<br>22 (3)<br>34 (3)<br>49 (3)<br>198 (3)	**152** (13)<br>**27** (7)<br>**34** (5)<br>**101** (5)<br>*49 (4)*<br>*255 (4)*<br>*22 (3)*	152 (16)<br>27 (13)<br>200 (10)<br>34 (9)<br>225 (9)	152 (9)<br>101 (4)<br>22 (3)<br>27 (3)<br>49 (3)	**152** (15)<br>**27** (10)<br>**34** (8)<br>200 (8)<br>**101** (6)<br>119 (6)<br>225 (6)	152 (18)<br>34 (16)<br>200 (15)<br>27 (12)<br>225 (11)	1<br>3
PF00141<br>1PA2, A<br>250<br>0.57	1983<br>442	97 (9)<br>44 (7)<br>48 (7)<br>176 (6)<br>49 (5)<br>91 (5)<br>41 (4)<br>45 (3)	**97** (10)<br>**44** (9)<br>**48** (9)<br>**176** (9)<br>**91** (8)<br>45 (6)<br>**49** (6)<br>**41** (5)<br>50 (5)<br>46 (5)	97 (16)<br>91 (14)<br>176 (14)<br>44 (13)<br>48 (12)<br>41 (10)	97 (9)<br>44 (8)<br>48 (8)<br>176 (8)<br>91 (7)	**97** (15)<br>**176** (14)<br>**48** (13)<br>**91** (13)<br>**44** (12)<br>**41** (10)<br>**49** (10)	97 (25)<br>176 (21)<br>45 (20)<br>48 (20)<br>91 (20)<br>44 (18)	2<br>2
PF00248<br>1GVE, A<br>322<br>0.41	4059<br>281	161 (6)<br>121 (5)<br>1 (4)<br>125 (3)<br>265 (3)<br>284 (3)<br>290 (3)	**161** (6)<br>1 (5)<br>121 (5)<br>**125** (5)<br>284 (5)	161 (10)<br>198 (8)<br>121(/)<br>125 (7)<br>284 (7)	161 (8)<br>125 (6)<br>84 (5)<br>115 (5)<br>121 (5)<br>198 (5)	198 (21)<br>115 (15)<br>84 (12)<br>**125** (10)<br>**161** (10)	198 (39)<br>115 (28)<br>84 (20)<br>145 (15)<br>43 (14)<br>125 (13)<br>152 (13)	0<br>3
PF00331<br>1R85, A<br>361<br>0.32	503<br>192	135 (5)<br>103 (4)<br>116 (3)<br>165 (3)	**135** (7)<br>**103** (5)<br>**116** (5)<br>*108 (4)*<br>*183 (4)*	108 (11)<br>103 (9)<br>135 (9)<br>183 (8)<br>116 (7)	103 (9)<br>108 (9)<br>135 (8)<br>116 (7)<br>175 (6)<br>183 (6)	**103** (17)<br>183 (17)<br>**116** (16)<br>108 (15)<br>**135** (15)	103 (32)<br>108 (26)<br>171 (26)<br>183 (26)<br>175 (24)<br>135 (23)	0<br>3
PF00550<br>1T8K, A<br>69<br>0.20	7380<br>512	34 (12)<br>43 (8)<br>6 (4)<br>50 (4)	34 (14)<br>43 (11)<br>6 (6)<br>57 (5)<br>*50 (4)*	34 (18)<br>43 (18)<br>6 (11)<br>50 (11)	-	-	34 (5)<br>6 (3)<br>43 (3)	0<br>2
PF00557<br>1QXY, A<br>239<br>0.53	2749<br>188	75 (8)<br>169 (5)<br>62 (4)<br>94 (4)<br>176 (4)<br>178 (4)	**75** (9)<br>**94** (7)<br>169 (6)<br>**62** (4)<br>176 (4)<br>**177** (4)<br>178 (4)	75 (12)<br>94 (11)<br>169 (7)<br>177 (7)<br>62 (6)<br>163 (6)<br>228 (6)	75 (7)<br>169 (6)<br>62 (4)<br>94 (4)<br>176 (4)<br>178 (4)	**75** (10)<br>**94** (9)<br>**177** (8)<br>27 (6)<br>20 (5)<br>**62** (5)	94 (15)<br>177 (15)<br>27 (13)<br>75 (12)<br>228 (12)	2<br>12
PF05199<br>1JU2, A<br>155<br>0.34	1157<br>257	361 (9)<br>455 (7)<br>359 (3)	**361** (13)<br>**455** (9)<br>*362 (4)*<br>*474 (4)*<br>*359 (3)*<br>*364 (3)*	361 (20)<br>455 (13)<br>364 (8)<br>399 (8)<br>359 (6)<br>474 (6)	361 (5)<br>455 (4)<br>359 (3)	**361** (13)<br>**455** (9)<br>420 (4)<br>359 (3)<br>457 (3)	361 (21)<br>455 (13)<br>359 (6)<br>362 (5)<br>420 (5)	0<br>0

Legend see next page

Column 1 lists the PFAM-number, the pdb-code of a representative protein and the symbol for the chain, the length of the MSA and the maximal *U(k, l)*-value determined in *H2r_filt_100*. The second column lists the number of sequences constituting the PFAM and the number of sequences after filtering with default parameters. The following 6 columns list residue positions as occurring in the related protein structure and their *conn(k)*-value for different settings of *HSRPs* used for computation. The last column lists the number of additional residues being *distinguished* in *H2r_filt_75* or *H2r_filt_60*, respectively. Residues having highest *conn(k)*-values both in the *HSRPs* = 75 and *HSRPs* = 2.5‰ experiment were printed in bold. A number given in brackets is a MSA position not occurring in the related protein. Residues printed italics were not *distinguished* in the related experiment.

Additional Table 6 - *conn(k)*-values for residues of 1QGN resulting from different numbers of high scoring residue pairs

#	***HSRPs***					
	**50**	**75**	**100**	**150**	**375**	**750**
388	8 (1)	10 (1)	10 (1)	11 (2)	14 (6)	17 (12)
268	5 (2)	7 (2)	9 (2)	13 (1)	23 (1)	37 (2)
305	4 (3)	6 (3)	8 (3)	10 (3)	22 (2)	38 (1)
386	4 (3)	6 (3)	6 (5)	9 (4)	18 (4)	35 (3)
393	5 (2)	6 (3)	7 (4)	9 (4)	12 (8)	19 (11)
308	3 (4)	5 (4)	5 (6)	7 (6)	14 (6)	21 (9)
391	3 (4)	5 (4)	6 (5)	8 (5)	13 (7)	19 (11)
86	2 (5)	4 (5)	4 (7)	6 (7)	9 (10)	13 (14)
402	2 (5)	4 (5)	4 (7)	8 (5)	18 (4)	27 (6)
387				5 (8)	21 (3)	34 (4)
389	3 (4)		5 (6)	6 (7)	9 (10)	19 (11)
289			4 (7)	7 (6)	16 (5)	33 (5)
406					13 (7)	26 (7)
168				3 (9)	13 (7)	24 (8)
267			3 (8)		12 (8)	19 (11)
288					7 (12)	26 (7)
111						20 (10)
354			4 (7)			20 (10)

Column 1 lists the residue number. The following columns give *conn(k)*-values and their ranks (in brackets) resulting from the specific number of *HSRPs* used for determination. These results were computed without adding pseudo counts.

Additional Table 7 - Composition of clusters for 11 datasets representing Cys_Met_Meta

Res #	80	86	99	107	**108**	**110**	111	113	114	116	122	141	155	163	164	182	197	**207**	209	211	212	**236**	238	239	245	258	**261**
$f_{max}(a_i)$	85	48	47	31	**100**	**96**	21	82	94	30	40	94	50	87	82	32	22	**99**	67	87	87	**100**	89	60	48	89	**100**
**1**		1<br>2			**1**		1		1				2	3<br>2			2		1	4<br>3	4<br>4		3<br>2	1		4<br>2	**134**
**2**		1			**1**		1	1<br>2		1			2	3	1	2	2			1<br>5	1<br>5	**1**	3	1	1	1<br>2	**13**
**3**		1<br>4	1<br>2		**1**		1			1	1		2				2		1<br>2					1	1		**1**
**4**	4	1<br>4			**1**		1		1		1		2	4<br>2	3		2		1				4<br>3	1			**14**
**5**		1							1				2	4<br>2	3<br>2	2		**1**		3<br>3	3<br>6		4<br>2	1		3<br>2	**134**
**6**		1<br>2				**4**	4						2<br>2	3<br>2		2	2	**1**		1<br>4	1<br>3		3<br>2	1		1<br>2	**13**
**7**		1		1	**1**								2	3	1<br>2	2		**1**		1<br>4	1<br>5		3	1		1<br>2	**13**
**8**	3<br>2	1<br>4							1				2<br>2	3<br>3	4	2	2		1				3<br>3	1			**13**
**9**		1			**1**								2	4<br>2	3<br>3		2	**1**		3<br>2	3<br>3		4<br>2	1			**14**
**10**		1			**1**							1	2<br>2	4<br>2	3<br>2	2	2	**1**		4<br>3	3<br>4		4<br>2	1			**14**
**11**		1			**1**							1	2	3<br>2	1		2	**1**		1<br>5	1<br>5		3<br>2	1		1<br>2	**13**
**Min**	1	1	2	1	**C**		1	2	1	1	1	1	1	1	1	1	1	**C**	1	2	3		1	1	1	1	**C**
**Max**	2	4	2	1	**C**		1	2	1	1	1	1	2	3	3	1	1	**C**	2	5	6		3	1	1	2	**C**
**Sum**	2	11	1	1			5	1	4	2	2	2	11	10	8	6	9		4	8	8		10	11	2	6	
**PFAM**	1		1				4	1	2	2	2								3						2	2	
**FFTNS**	1			1			1		2										1							3	
**LINSI**												2														1	

Res #	266	267	268	**272**	289	290	**292**	299	305	308	**313**	354	386	387	388	389	**390**	391	393	399	402	403	404	416	**423**	425
$f_{max}(a_i)$	83	43	61	**100**	38	24	**98**	46	60	38	**100**	50	46	23	62	56	**96**	47	52	87	42	71	82	88	**100**	81
**1**	3<br>2	1<br>2	1<br>6		1<br>2		**4**	1	1<br>4	1<br>4	**1**	1<br>2	1<br>4	4	1<br>10	1<br>3	**13**	1<br>3	1<br>5		1<br>3	4	4<br>2		**4**	4<br>2
**2**		1<br>2	1<br>5	**1**	1<br>3				1<br>4	1<br>3	**1**	1<br>2	1<br>4		1<br>8	1<br>4	**1**	1<br>4	1<br>5		1<br>3	1<br>3	1<br>3			1<br>2
**3**		1<br>2	1<br>8		1<br>4			1	1<br>8	1<br>5	**1**	1<br>4	1<br>6		1<br>10	1<br>5	**1**	1<br>6	1<br>6		1<br>4					
**4**	4<br>2	1<br>2	1<br>7		1<br>2			1	1<br>6	1<br>5	**1**	1<br>3	1<br>6		1<br>10	1<br>3	**14**	1<br>5	1<br>6		1<br>4	3	3<br>2			
**5**	4<br>2	1<br>2	1<br>4						1<br>4	1<br>3	**1**	1	1<br>4	1	1<br>9	1<br>3	**14**	1<br>3	1<br>5	3<br>3	1<br>3	3	3<br>4	3	**3**	3<br>2
**6**	3<br>2	1<br>3	1<br>6			4	**1**		1<br>4	1<br>4	**1**	1	1<br>4		1<br>9	1<br>4	**13**	1<br>4	1<br>6		1<br>6					1<br>2
**7**		1<br>2	1<br>5				**1**		1<br>3	1<br>3	**1**	1	1<br>4	1	1<br>9	1<br>4	**1**	1<br>4	1<br>5	1<br>3	1<br>5	1<br>2	1<br>2	1	**1**	1<br>2
**8**	3<br>2	1<br>2	1<br>7						1<br>5	1<br>4	**1**	1<br>3	1<br>6		1<br>9	1<br>5	**13**	1<br>3	1<br>6		1<br>4		4			
**9**	4<br>2	1<br>2	1<br>6	**1**	1				1<br>4	1<br>4	**1**	1	1<br>4	1	1<br>9	1<br>3	**14**	1<br>4	1<br>5	3<br>3	1<br>4	3<br>2	3<br>3		**3**	3<br>2
**10**	4<br>2	1<br>2	1<br>6		1				1<br>4	1<br>4	**1**	1	1<br>4		1<br>9	1<br>3	**14**	1<br>4	1<br>5	3<br>3	1<br>3	3	3<br>5		**3**	3<br>2
**11**	3	1<br>2	1<br>4		1				1<br>4	1<br>3	**1**	1	1<br>4	1	1<br>8	1<br>4	**13**	1<br>3	1<br>6	1<br>3	1<br>5	1	1<br>5		**1**	1<br>2
**Min**	1	2	4	**C**	1	1	**C**	1	3	3	**C**	1	4	1	8	3	**C**	3	5	3	2	1	1	1	**C**	2
**Max**	2	2	8	**C**	3	1	**C**	1	8	5	**C**	3	6	1	10	5	**C**	5	6	3	6	3	5	1	**C**	2
**Sum**	8	11	11		7	1		3	11	11		11	11	5	11	11		11	11	5	11	8	9	2		8
**PFAM**								3						1						0						
**FFTNS**						1								2						2				2		
**LINSI**														2						3						

For each of the MSAs 1 - 11 given in Additional Table 4, the residues constituting clusters were determined and listed. The first line gives the residue number as deduced from 1QGN. The second line lists the maximal frequency $f_{max}(a_i)$ of any amino acid occurring in the respective column of the MSA. The following lines, labelled 1 – 11, represent the composition of the clusters. For each residue, two numbers are given. The upper one is the number of the cluster, the residue *k* was an element of. The lower number gives the *conn(k)*-value, if *conn(k)* > 1. The lines labelled "Min" or "Max" list the lowest and the highest *conn(k)*-value. Line "Sum" gives the number of clusters the residue was an element of. Lines PFAM, FFTNS, and LINSI give the number of clusters the residue belonged to for those specific datasets. Strictly conserved residues are printed bold. 12 assignments that could be identified as being specific for a dataset or a method of MSA generation were labelled with a grey background. **Example**: The second column has to be read as follows: At residue 80, an amino acid occurred with a maximal frequency of 85%. For dataset 4, this residue was element of cluster 4 with a *conn(k)*-value of 1, in dataset 8 it belonged to cluster 3 and had a *conn(k)*-value of 2. The minimal *conn(k)*-value of this residue was 1, the maximal one was 2. In summary, residue 80 was element of 2 clusters (Sum value), 1 cluster originated from the PFAM datasets, and the other one was generated by using the FFTNS option of MAFFT.

Additional Table 8 - A comparison of the outcome of three algorithms for predicting residue correlations in *H2r_filt_100*

PFAM PDB, chain	*H2r*	CorrMut	P2PConPred
PF000026 1WKR, A	[209] (5), **236** (6), 291 (5)	(94,242), (219,242), (52,139), (54,132), (**236**,263)<br>(218,243), (218,299), (219,242), (218,245), (218,275)	(247,290), (48,290), (48,247), (137,287), (48,201)
PF000067 1JFB, A	283 (9), 287 (8), 289 (5), 291 (9), 314 (5)	(236,298), (157,231), (172,255), (260,373), (255,279)<br>(232,255), (236,298), (99,248), (172,255), (128,319)	No corr. det.
PF00068 1G4I, A	11 (6), 41 (9), 50 (5), 77 (5), 85 (5), 110 (6), 115 (5)	(28,76), (59,82), (82,93), (82,89), (28,60)<br>(28,76), (28,60), (82,93), (82,89), (59,82)	(45,84), (44,51), (51,27), (51,29), (29,96)
PF000089 1HJ9, A	**27** (7), 34 (5), **101** (5), 152 (13)	(70,120), (106,119), (114,135), (24,106), (**101**,164)<br>(103,240), (**27**,103), (24,106), (103,130), (106,114)	No corr. det.
PF00141 1PA2, A	41 (5), 44 (9), 45 (6), 46 (5), 48 (9), 49 (6), 50 (5), 91 (8), 97 (10), 176 (9)	(81,259), (268,293), (137,221), (81,256), (105,226)<br>(219,262), (68,219), (105,226), (70,208), (147,297)	No corr. det.
PF00248 1GVE, A	1 (5) 121 (5), 125 (5), 161 (6), 284 (5)	(56,103), (58,90), (58,102), (89,105), (58,101)<br>(58,102), (89,105), (58,90), (105,142), (58,76)	No corr. det.
PF00331 1R85, A	103 (5), 116 (5), 135 (7)	(151,226), (226,318), (167,226), (51,151) (51,250)<br>(69,193), (228,248), (167,251), (221,244), (223,251)	No corr. det.
PF00550 1T8K, A	6 (6), 34 (14), 43 (11), 57 (5)	(26,30), (26,72), (4,8), (62,63), (30,72)<br>(26,30), (4,8), (24,26), (62,63), (23,62)	(25,66), (13,45), (47,66), (47,61), (61,66)
PF00557 1QXY, A	62 (4), 75 (9), 94 (7), 169 (6), 176 (4), 177 (4) 178 (4)	(8,31), (165,211), (130,161), (**13**,205); (18,37)<br>(142,193), (11,204), (47,193), (172,204), (24,204)	(**13**,202), (214,240), (49,54), (231,234), (49, 214)
PF05199 1JU2, A	361 (13), 455 (9)	(29,191), (120,468), (157,347), (193,255), (265,325)<br>(241,414), (242,414), (392,414), (322,414), (103,312)	(404,443), (368,442), (442,483), (443,483), (394,443)

Column 1 lists the PFAM-number, the pdb-code of a representative protein and the chain symbol. Column 2 gives significant *conn(k)*-values deduced from the analysis of the respective PFAM by using *H2r*. The column CorrMut list five pairs ranking lowest on their Pearson correlation value (upper line) or their expected Pearson correlation value (lower line), respectively. The column P2P ConPred lists those 5 residue pairs having highest correlation scores. Residues predicted by two algorithms as being involved in correlations are printed bold. A residue given in brackets is a MSA position not occurring in the related protein.

Additional Table 9 - Analysis of ATP synthase ε subunit of *Escherichia coli*

#	Evidence	Z-score	*conn(k)*	*max U(k, l)*	*max MI/H(k, l)*
65	IDLM	4.5	6	0.35 (5)	0.42 (5)
81	LMP	4.5	7	**0.36 (2)**	**0.45 (2)**
16	AGM	4.0		0.33 (17)	0.40 (17)
70	IDLM	3.7		0.35 (4)	0.43 (4)
72	nr		15	**0.37 (1)**	**0.45 (1)**
73	nr			**0.37 (1)**	**0.45 (1)**
12	nr		5	0.35 (3)	0.43 (3)
22	nr			0.35 (6)	0.42 (6)
46	L	3.5		**0.36 (2)**	**0.45 (2)**
31	DIM	3.4		0.35 (7)	0.42 (7)
77	DGLM	3.4		-	-
54	nr	3.2			
82	I	2.9			
85	IL	2.8			
10	AGMI	2.4			
63	DGLMP	2.4			
43	LI	1.8			

The MSA constituting PF02823 was analysed by using *H2r*. Residue numbers resulting from a projection of the MSA onto pdb structure 1AQT are listed in column 1. Column 2 lists evidence for effects of mutations *in vitro* as compiled in [3]. Functions affected are indicated as follows: A, assembly of ATP synthase; D, inhibition of ATPase; G, cell growth; I, inhibition of ATPase by ε subunit; L, stimulation of ATPase by lauryl dimethylamine oxide; M, membrane bound ATPase activity; P, ATP driven proton translocation in membranes. Changes greater 20% were considered significant. nr: none reported. The column "Z-score" lists data that have been reported in [3]. Column conn(k) lists all values ≥ 5, columns *max U(k, l)* and *max MI/H(k, l)* list the maximal values and their ranks for the considered residue. Predictions resulting from the largest 4 *U(k, l)*-values were printed in bold.
